# Room-Temperature Phosphorescence Vapochromism through Conformational Control

**DOI:** 10.1021/jacs.5c07339

**Published:** 2025-08-26

**Authors:** Andrea Fermi, Sapna Gahlot, Eric Jorand, Simone d’Agostino, Yasi Dai, Fabrizia Negri, Corinne Moustrou, Marc Gingras, Paola Ceroni

**Affiliations:** † Dipartimento di Chimica “Giacomo Ciamician”, 9296 Alma Mater Studiorum−Università di Bologna, Via Gobetti 85, 40129 Bologna, Italy; ‡ 128791 Aix-Marseille Université, CNRS, CINaM, 13288 Marseille, France

## Abstract

Solid-state room-temperature phosphorescence is rarely observed in organic molecules, and the modification of its color upon application of physical or chemical stimuli is hardly achieved. In this work, we demonstrate that a decorated persulfurated benzene shows reversible phosphorescence switching in the solid state, as a consequence of conformational changes induced by inclusion of solvent molecules. Quantum-chemical calculations suggest that the luminescence changes are due to emission from triplet states of different character. These results display the close relationship between structural factors and phosphorescence in solid state emitters, representing a valuable benchmark for the design of all-organic luminescent sensors with the desired photophysical properties.

## Graphical abstract

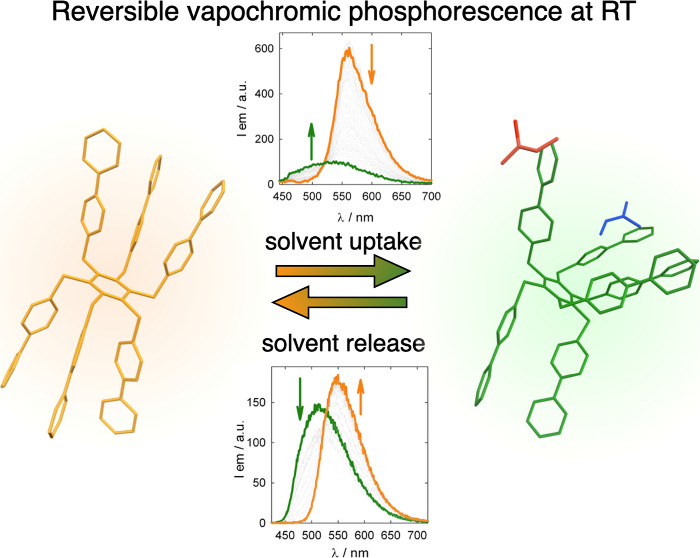

Due to their countless applications in light generation,^1–3^ molecular and inorganic materials with tailored luminescent properties in the solid phase have been extensively studied in the last decades in crystal engineering.^4,5^ More recently, increasing attention has been devoted to materials able to show exalted room-temperature phosphorescence (RTP) in the solid state, a phenomenon most commonly detected in metal complexes.^6^ The same property is rather elusive for organic materials,^7,8^ since the population of the emitting triplet state cannot take advantage of heavy atoms able to induce spin–orbit coupling (SOC) and high intersystem crossing (ISC) rates.^8,9^ The design of organic materials showing high phosphorescence quantum yield in the solid phase is therefore limited by multiple chemical and structural factors, although the production could be achieved on a large scale (in contrast to their precious noble metal-based counterparts).^5,10–13^ As a consequence, the rational control of key luminescence parameters (i.e., energy, lifetime, quantum yield) is hardly realized, still making the development of stimuli-responsive organic triplet emitters highly challenging.^14,15^

Arylthio-substituted persulfurated benzenes (such as **A6-Tol**, Scheme 1) represent an intriguing class of solid-state RTP emitters that we pioneered in 2013.^16–22^ While the relationship between their luminescent properties and structural factors (such as intramolecular conformation,^13a,17^ polymorphism,^20^ aggregation^18,23^ ) or physical stimuli (such as temperature^17^) have been described, the phosphorescence tuning via application of a chemical stimulus has not been observed within this class of compounds. Persulfurated benzenes and related compounds have also displayed intriguing properties as solid-state clathrates in the presence of solvent molecules,^24–27^ demonstrating their structural adaptability to the inclusion of several guests.

**Scheme 1:**
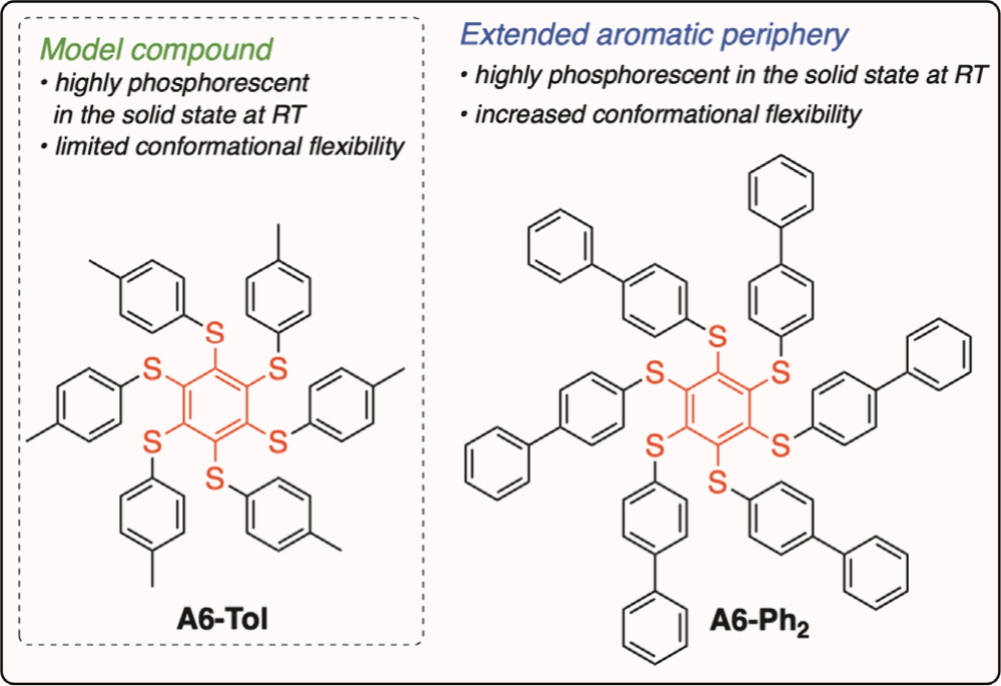
Aryl-Substituted Persulfurated Benzenes Taken into Exam

In light of these aspects and the results collected by our groups on per­(phenylthio)­benzenes,^13a,16,17^ we rationally designed simple materials based on a structural elongation for enhancing supramolecular interactions and for exhibiting reversible structure-dependent RTP in the presence of guest molecules. For this novel class of solid optical sensors, we selected simple biphenylthio arms to achieve a structural balance that maximizes phosphorescence and its effective control upon supramolecular inclusion.

Here, we describe how the phosphorescence of solid **A6-Ph**_**2**_ (Scheme 1) can be switched through the conformational change induced by the uptake of solvent molecules (i.e., vapochromism^28,29^ ), a property that has been exclusively observed for inorganic phosphors at RT.^30–32^

We therefore present a class of fully organic, phosphorescent stimuli-responsive materials, obtained through simple and scalable protocols, thus demonstrating a new approach for solid-state optical sensing devices based on the rational control of intramolecular conformations.

Compound **A6-Ph**_**2**_ can be prepared through nucleophilic aromatic substitutions onto perhalogenobenzene, in the presence of an excess arylthiols and a base (e.g., K_2_CO_3_, *t*-BuOK, or NaH) in polar aprotic solvents, such as DMF (see the Supporting Information, SI).^17,25,33,34^

The profile of the absorption spectrum of compound **A6-Ph**_**2**_ in CH_2_Cl_2_ between ca. 340 and 450 nm is similar to that recorded for model compound **A6-Tol**,^16^ while a major peak attributed to parent **Ph**_**2**_ **-SMe** is detected at 288 nm (Figure 1A and Figure S11). The increase in the extension of the peripheral π-system is therefore not significantly affecting the energy of the lowest-lying electronic transition. The same considerations hold by taking into account the luminescence properties in diluted rigid matrix at 77 K, where the emission spectra of **A6-Tol** and **A6-Ph**_**2**_ share comparable profiles, lifetimes in the order of 10^–3^ s and emission quantum yield (Figure 1B and Table 1). Similarly to **A6-Tol**, no significant emission (Φ_em_ < 10^–3^) has been detected for **A6-Ph**_**2**_ in air-equilibrated or deoxygenated solutions at RT:^16^ this behavior can be ascribed to the efficient nonradiative deactivation of the excited state in low viscosity media at RT.^35–40^

**Figure 1:**
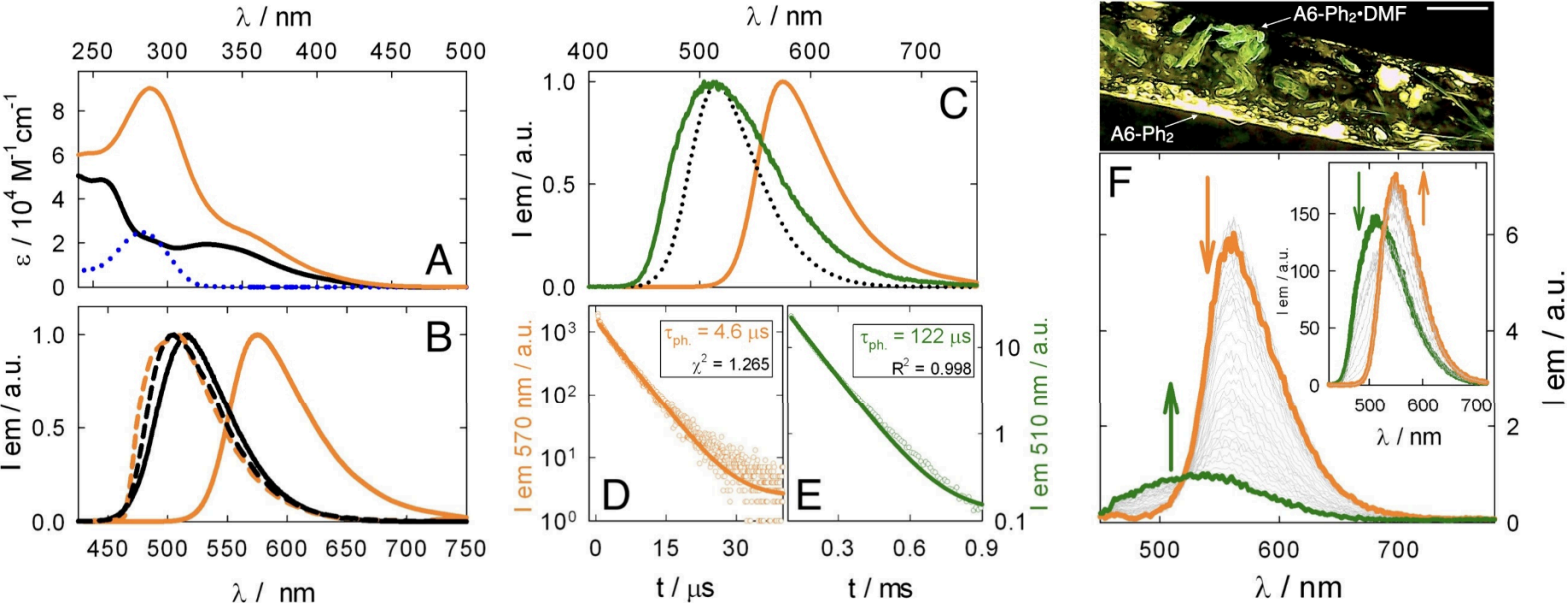
(A) Absorption spectra in CH_2_Cl_2_ at RT for **A6-Tol** (black line), **A6-Ph_2_** (orange) and Ph_2_-SMe (dotted line). (B) Normalized emission spectra of **A6-Tol** (black line) and **A6-Ph_2_** (orange) in the solid state at RT and in a rigid matrix at 77 K (dashed lines; CH_2_Cl_2_:CH_3_OH, 1:1 v/v). (C) Normalized emission spectra of solid **A6-Ph_2_** (orange), **A6-Ph_2_·DMF** (green), and **A6-Tol** (dotted line) at RT. (D, E) Emission decays for solid **A6-Ph_2_** and **A6-Ph_2_·DMF**, respectively, at RT. Monoexponential fitting curves are also shown. (F) Emission changes in solid **A6-Ph_2_** upon exposure to DMF vapors at RT. Inset: reversible release of DMF molecules by solid **A6-Ph_2_·DMF** in air. Top: micrograph of a glass pipette showing the phosphorescence of the pure and solvated form (scale bar: 1 mm). 340 **<** λ_ex_ **<** 380 nm.

**Table 1:** Photophysical Data Collected under Different Experimental Conditions

		Emission					
		solid, RT			CH_2_Cl_2_:CH_3_OH (1:1, v/v), 77 K		
Compound	Absorption, CH_2_Cl_2_, RT: λ (nm)/ε (M^–1^ cm^–1^)	λ_max_ (nm)	τ (μs)	Φ	λ_max_ (nm)	τ (ms)	Φ
**A6-Tol** ^a^	328/19400	517	3.0	1.0	505	4.0	1.0
**Ph**_**2**_ **-SMe**	283/24800	not luminescent			495^b^	214^b^	n.d.
**A6-Ph**_**2**_	343/27700	575	4.6	0.8	510	7.5	1.0
**A6-Ph**_**2**_ **·DMF**		504	122	n.d.^c^			

a Data from ref 17.

b Phosphorescence data.

c Data not determined due to the instability of the samples.

More interestingly, the luminescence of pure **A6-Ph**_**2**_ in the solid state at RT is remarkably different in respect to those usually observed for similar derivatives, such as **A6-Tol**:^16,17^ the emission of **A6-Ph**_**2**_ is significantly red-shifted, peaking at λ_max_ = 575 nm (Figure 1B). Such changes have been rarely observed for this class of compounds^20^ or for sulfurated derivatives of dicyanobenzene.^41,42^ Importantly, the emission lifetime (τ_ph._ = 4.6 μs; Figure 1D) indicates radiative deactivation of a triplet excited state at RT. The remarkable emission quantum yield (Φ_ph._ = 0.8) makes compound **A6-Ph**_**2**_ another attractive example of an RT organic solid-state emitter within this family of compounds.

Comparison between absorption and excitation spectra collected from solid samples of **A6-Tol** and **A6-Ph**_**2**_ (Figure S13A,B) shows a significant shift to higher wavelengths for the latter. This aspect remarks differences in the absorption energies of the solid phases that are often attributable to structural factors, such as packing geometry, arrangements, and molecular conformation adopted in their crystal lattices.^5^

The structural analysis of bright orange polycrystalline samples of **A6-Ph**_**2**_ was carried out through powder X-ray diffraction (PXRD). The six biphenylthio substituents attached to the benzene core reveal an *aaabbb* pattern (*a* and *b* indicating substituents positions above and below the plane of the central ring, respectively), resulting in a chairlike conformation (Figure 2A and Table S1).^43^ No specific intermolecular interactions are observed in the lattice of **A6-Ph**_**2**_; crystal cohesion is due to dispersion forces and by molecules packing mainly by shape factor,^44^ i.e. interlocking themselves in a slipped columnar fashion (Figure 2C).

**Figure 2:**
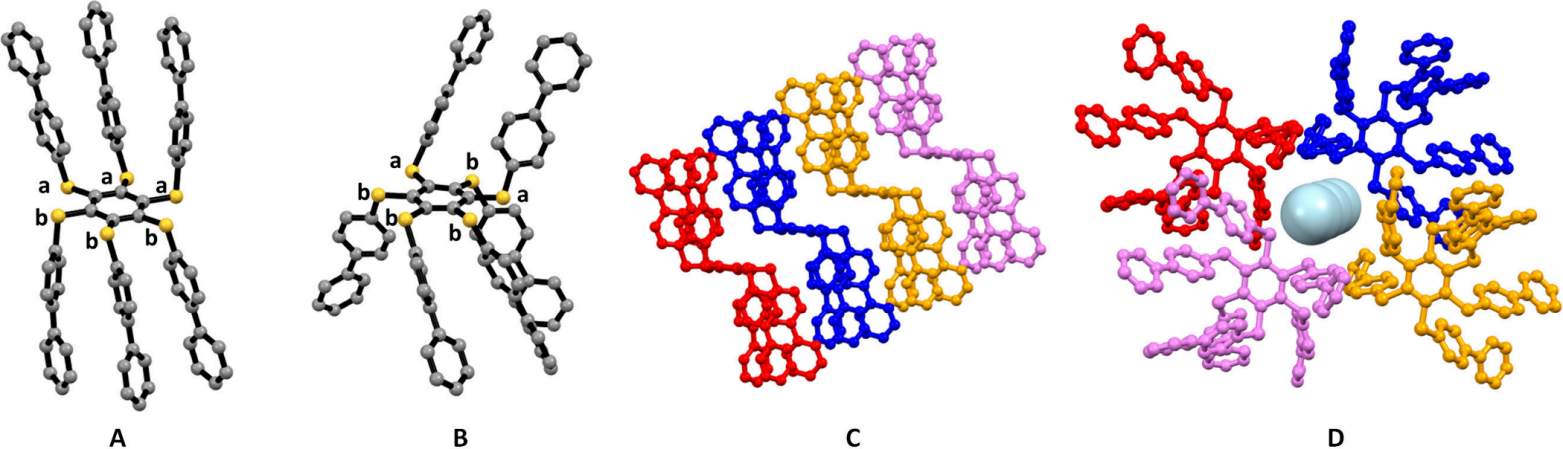
Molecular structures of **A6-Ph**_**2**_ (A) and **A6-Ph**_**2**_ **·X** (B), as determined from SC-XRD. Letters in lowercase indicate the orientations of the peripheral substituents with respect to the central benzene unit (a: above; b below; solvent molecules are not shown). C: columnar stacking detected in **A6-Ph**_**2**_. D: solvent pocket in **A6-Ph**_**2**_ **·X** formed by four adjacent molecules, with solvent molecules shown as light-blue spheres; H_CH_ is omitted for clarity.

Importantly, we noticed that the crystalline materials obtained from DMF and dimethylacetamide (DMA) solutions exhibited similar powder XRD diffraction patterns and distinct from those observed for the as-synthesized **A6-Ph**_**2**_ (Figure S7). The thermogravimetric analysis (TGA) on the crystals grown in DMF and DMA indicated the presence of approximately 2–3 solvent molecules per each **A6-Ph**_**2**_ (Figure S10), highlighting that the compound crystallizes as a clathrate, as observed in similar materials.^24^ In contrast, TGA measurements on the as-synthesized material confirmed the absence of solvent molecules. Therefore, the crystalline materials could be formulated as **A6-Ph**_**2**_ in its solvent-free form and as **A6-Ph**_**2**_ **·X** (where X = DMF, DMA) when isolated as solvates. These observations were further validated by single crystal XRD analysis (SC-XRD, details in Table S1). Figure 2B displays the crystal structures as determined by SC-XRD data for **A6-Ph**_**2**_ **·X**: the substituents adopt an asymmetric conformation with an *ababbb* pattern.^20^ The crystal packing in **A6-Ph**_**2**_ **·X** highlights how the arrangement of **A6-Ph**_**2**_ molecules allows solvent molecules to fit into pockets (Figure 2D), thanks to weak hydrogen bond interactions between aromatic CH moieties from **A6-Ph**_**2**_ molecules and O atoms from the solvent [*d*(CH_ar_···O_C=O_) = 3.282(8)–3.547(9) and 3.32(1)–3.56(2) Å for **A6-Ph**_**2**_ **·DMF** and **A6-Ph**_**2**_ **·DMA**, respectively]. Both **A6-Ph**_**2**_ **·X** clathrates tend to easily lose solvent molecules when exposed to air at RT, as indicated by TGA analysis (Figure S10). By warming above 150 °C the original nonsolvated phase **A6-Ph**_**2**_ is obtained, as confirmed by powder XRD analysis (Figure S7).

The structural differences introduced in the solvated **A6-Ph**_**2**_ **·X** crucially impact the luminescent properties: compared to the solvent-free form, the isolated crystals of **A6-Ph**_**2**_ **·X** show a greenish phosphorescence reminiscent of model compound **A6-Tol** (Figure 1C and Table 1). The corresponding lifetime (τ_ph._ = 122 μs, Figure 1E) indicates phosphorescence emission from the solvated material.

**A6-Ph**_**2**_ was thus employed in vapochromism tests, monitoring luminescence changes upon exposure to solvent vapors (details in SI). When the solvent-free form was exposed to DMF vapors, an increase in emission intensity around 505 nm was detected, accompanied by the substantial decrease of the original emission. This trend highlights the conversion of the pure form to **A6-Ph**_**2**_·**DMF** (Figure 1F). Remarkably, the changes observed in the photophysical behavior are reversible: upon exposure to air, the so-obtained **A6-Ph**_**2**_ **·DMF** releases DMF molecules, restoring the original phosphorescence signal of solvent-free **A6-Ph**_**2**_ (Figure 1F, inset). Hence, solid compound **A6-Ph**_**2**_ can reversibly convert its phosphorescence emission as a consequence of the conformational changes induced by the inclusion/release of DMF molecules. On the other hand, such behavior has not been observed with chlorinated solvents such as CH_2_Cl_2_ and CHCl_3_ or with aromatic C_6_H_6_ (see Figure S20). Differences between the phosphorescence profiles of the pure and solvated forms are observed as well at low temperatures (Figure S15 and Table S2), indicating that the structural parameters of the two forms are not significantly affected upon increasing the rigidity of the lattice.

To understand the red-shifted emission of the solvent-free **A6-Ph**_**2**_ crystal compared to its solvated form, we performed quantum-chemical calculations on the isolated *aaabbb* and *ababbb* conformers as well as their corresponding aggregates. At the optimized ground-state geometry, the lowest triplet state of **A6-Ph**_**2**_ *aaabbb* has a dominant nπ* nature, while **A6-Ph**_**2**_ *ababbb* is predominantly ππ*.

At higher energies, another triplet state with a localized excitation on biphenyl branches emerges (hereafter indicated as Ph_2_* character, Table S4). Specifically, in isolated **A6-Ph**_**2**_, geometry optimization of the Ph_2_* triplet state places its emission energy at 2.26 eV, and its minimum 3.95 kcal/mol above the nπ* state in the *aaabbb* conformation, while it appears 12.2 kcal/mol above the ππ* in *ababbb* including two explicit DMF solvent molecules (Figure 3). Triplet-state geometry optimization predicts an **A6-Ph**_**2**_ *aaabbb* emission energy from the nπ* triplet of 2.30 eV, similar to that of **A6-Tol** (2.32 eV, Table S5), contradicting experimental data (Figure 1).^13a^ Investigations on dimer and trimer aggregates of conformer *aaabbb* of **A6-Ph**_**2**_ do not evidence excitonic interactions, while they suggest an inversion between the nπ* and the Ph_2_* triplet states (Figure 3 and Table S6). Similarly, QM/MM calculations suggest that this inversion takes place only in the *aaabbb* conformer (solvent-free crystals) and not in the *ababbb* conformer (solvated **A6-Ph**_**2**_), as a result of the closer packing of **A6-Ph**_**2**_ molecules in the former (Figure 3 and Table S7, SI).

**Figure 3:**
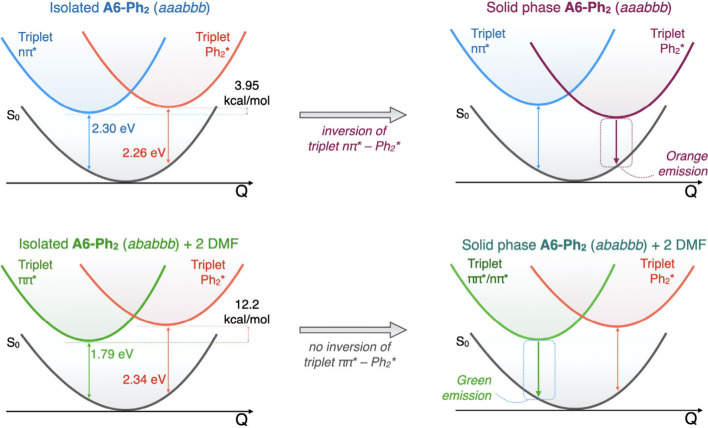
Schematic representation of the calculated potential energy profiles vs. the multidimensional displacement coordinate for the triplet excited states (nπ*/ππ* and Ph_2_* character) of **A6-Ph**_**2**_ (top: *aaabbb;* bottom: *ababbb*) as isolated/solvated molecules and as aggregates.

To investigate the reversible conformational change between the solvent-free and solvated crystals, we conducted molecular dynamics (MD) simulations (SI). Our results confirmed that conformational transitions in **A6-Ph**_**2**_ molecules, combined with the voids originated by the release of solvent molecules, led to the formation of different conformers during MD runs of up to 20 ns. Although this time scale is too short to definitively capture the *ababbb*-to-*aaabbb* transition observed for **A6-Ph**_**2**_—particularly given the influence of the solvent—these results strongly support the reversibility of the conformational changes.

Overall, these findings demonstrate a rationale to design semiflexible persulfurated benzenes and to achieve stimuli-responsive RTP material in the solid state, without using noble metal complexes. Inclusion of solvent molecules leads to significant conformational changes that affect the emission color and photophysical properties. Importantly, the vapochromic behavior at RT is indubitably accompanied by the reversible conversion between two conformations. Calculations suggest that the observed vapochromism is associated with a triplet emission that reflects the different nature of the two conformers: the red-shifted emission originates from the Ph_2_* triplet state of *aaabbb* conformers in solvent-free **A6-Ph**_**2**_, while the core centered ππ* state is responsible for emission in the solvated crystal, as observed in **A6-Tol** and similar derivatives. Thus, we open new avenues for the design of novel and scalable all-organic sensing devices and optoelectronic materials that are highly effective in the solid state by RTP.

## Supplementary Material



## Notes

Synthetic details, XRD data, thermogravimetric analysis, photophysical characterization, and computational details (PDF)
