# Supplementary material for: Room-Temperature Phosphorescence Vapochromism through Conformational Control
Source: J Am Chem Soc. 2025 Aug 26;147(36):32309–14. doi: 10.1021/jacs.5c07339 (PMC12426879; doi:10.1021/jacs.5c07339)
Supplement: Supplementary file 1 [file ja5c07339_si_001.pdf]

SUPPORTING INFORMATION

Room-Temperature Phosphorescence Vapochromism Through Conformational Control

Andrea Fermi,[a] Sapna Gahlot,[b] Eric Jorand,[b] Simone d'Agostino,*[a] Yasi Dai,[a] Fabrizia Negri,*[a] Corinne Moustrou,[b] Marc Gingras*[b] and Paola Ceroni[a]*

[a] Dipartimento di Chimica "Giacomo Ciamician", Alma Mater Studiorum – Università di Bologna, Via Gobetti 85, 40129 Bologna, Italy

[b] Aix-Marseille Université, CNRS, CINaM, 13288 Marseille, France

Table of Contents

SYNTHETIC PROCEDURES 2
NMR ANALYSES 4
HRMS ANALYSES 6
XRD ANALYSES 7
TGA ANALYSES 11
PHOTOPHYSICAL ANALYSES 12
COMPUTATIONAL DETAILS 17
SUPPORTING REFERENCES 31

SYNTHETIC PROCEDURES

General information

^{1}H-NMR and ^{13}C-NMR spectra were recorded on Varian Mercury 400 MHz spectrometer or on a Bruker Avance 600 spectrometer. The chemical shifts (δ) for ^{1}H are given in ppm relative to residual signals of the solvents ($CDCl_3$: δ = 7.26 ppm). Data are reported as follows: chemical shift (δ), multiplicity (s = singlet, d = doublet, t = triplet, q = quartet, dd = doublet of doublets, m = multiplet, etc.), coupling constants (Hz). The chemical shifts (δ) for ^{13}C are given in ppm relative to residual signals of the solvents and tetramethylsilane (TMS) at 0 ppm ($CDCl_3$: δ = 77.16 ppm). GC-MS spectra were taken by EI ionization at 70 eV on a Hewlett-Packard 5971 with GC injection. Chromatographic purifications were done with 240-400 mesh silica gel. HRMS analyses were performed on Waters Xevo G2-XS QTof, ESI$^+$, cone voltage 40 V, capillary 3 kV, source temperature 120 °C. All reactions were set up under an argon or nitrogen atmosphere in oven-dried glassware using standard Schlenk techniques. Unless specified, other anhydrous solvents were used without further purifications. All reagents were purchased from commercial suppliers (Sigma-Aldrich, Alfa Aesar, Fluorochem, TCI, etc.) and used without further purification unless specified. The synthesis of **A6-Tol** has been reported elsewhere.[1–3]

Synthesis of Ph_2-SMe {[1,1'-biphenyl]-4-yl(methyl)sulfane}

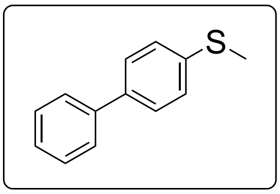

To a solution of [1,1′-Biphenyl]-4-thiol (50 mg, 0.268 mmol, 1.0 eq) and K_2CO_3 (186 mg, 1.342 mmol, 5 eq) in acetonitrile (0.6 mL), iodomethane (0.084 mL, 0.342 mmol, 5.0 eq) was added, under Ar atmosphere. The mixture was then stirred at 21°C for 24 hours. After 24 hours, the solvent was removed under vacuum and an aqueous solution of HCl (2M, 5 mL) was added. The aqueous layer was extracted with chloroform (5x5 mL). The organic layer was then dried with $MgSO_4$ and filtrated. The solvent was then evaporated under reduced pressure to afford the product (53.2 mg, 99% yield) as a white powder. NMR characterization (^{1}H and ^{13}C) is in agreement with literature data.[4,5]
^{1}H NMR (600 MHz, $CDCl_3$, ppm): δ = 7.58 (d, 2 H, J = 7.6 Hz), 7.53 (d, 2 H, J = 7.5 Hz), 7.43 (t, 2 H, J = 7.4 Hz), 7.35-7.33 (m, 3 H), 2.53 (s, 3 H). **^{13}C NMR** (151 MHz, $CDCl_3$, ppm): δ = 140.7 (1 C), 138.2 (1 C), 137.7 (1 C), 129.0 (2 C), 127.7 (2 C), 127.4 (1 C), 127.1 (2 C), 127.0 (2 C), 16.1 (1 C).

Synthesis of A6-Ph_2 [1,2,3,4,5,6-hexakis([1,1'-biphenyl]-4-ylthio)benzene]

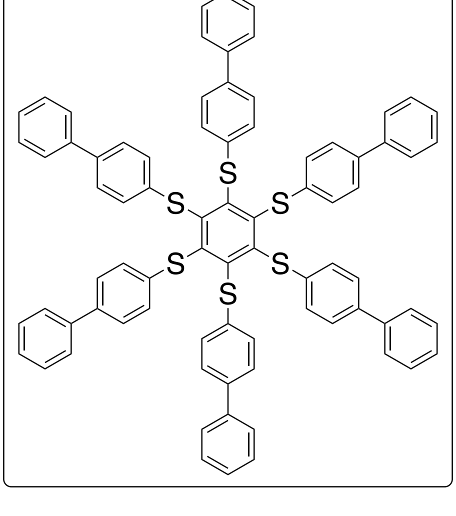

In an oven dried sealed tube, purged with argon, hexachlorobenzene was added (20.6 mg, 0.072 mmol, 1.0 eqv), together with dried potassium carbonate (87.4 mg, 0.632 mmol, 9 eqv), biphenyl-4-thiol (118.7 mg, 0.637 mmol, 9 eqv) in dry DMF (1 mL, dried on molecular sieves 3Å). Ar was bubbled through the mixture for 5-10 min in the reaction tube. The tube was then sealed and the reaction was stirred at 90° C for 40 hrs. Most DMF was then removed on a rotary evaporator under reduced pressure. The reaction mixture was extracted with H_2O (35mL) and toluene (4×35mL). The collected organic phase was dried over anhydrous $MgSO_4$, then evaporated. The crude mixture was triturated with ethanol (30 mL) at RT (25-28° C) and was filtered to get 68.8 mg of **A6-Ph_2**, as an orange solid (yield = 82%). R_f = 0.40 (toluene/cyclohexane 3:7); m. p.: 250-252°

C. For XRD analysis, solvent-free crystals were obtained from slow evaporation of a solution in boiling toluene; solvated forms **A6-Ph$_2$•DMF** and **A6-Ph$_2$•DMA** were obtained from slow evaporation at RT of solutions in dimethylformammide and dimethylacetamide, respectively. The solvated form obtained from slow evaporation of solutions of **A6-Ph$_2$** in hot pyridine has been prepared as yellow needle-like crystals (XRD analysis not available due to instability of the sample).

^{1}H NMR (400 MHz, $CDCl_3$, ppm): δ = 7.07 (d, *J* = 6.3 Hz, 12 H), 7.29-7.31 (m, 18 H), 7.37-7.41 (m, 24 H).
^{13}C NMR (101 MHz, $CDCl_3$, ppm): 126.95 (12 C), 127.45 (6 C), 127.69 (12 C), 128.82 (12 C), 128.94 (12 C), 136.68 (6 C), 139.27 (6 C), 140.17 (6 C), 148.07 (6 C).
HRMS (ESI$^+$, m/z)**:** requires 1182.2550 for $C_{78}H_{54}S_6$; found: 1183.2644 (M-H$^+$).

NMR ANALYSES

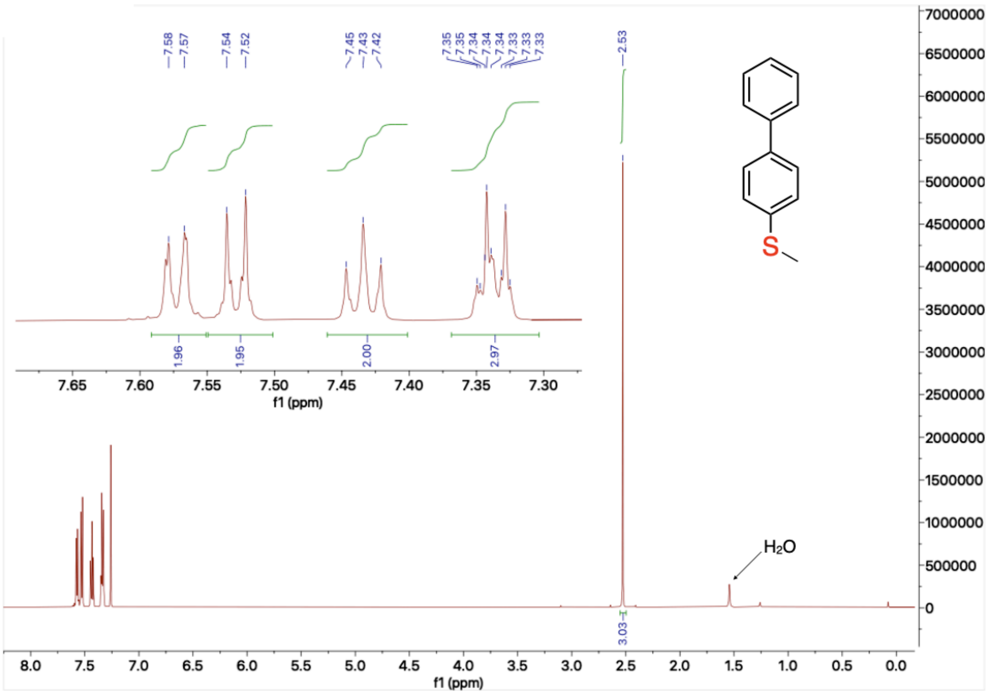

Figure S 1. ^{1}H NMR of **Ph$_2$-SMe** (600 MHz, $CDCl_3$).

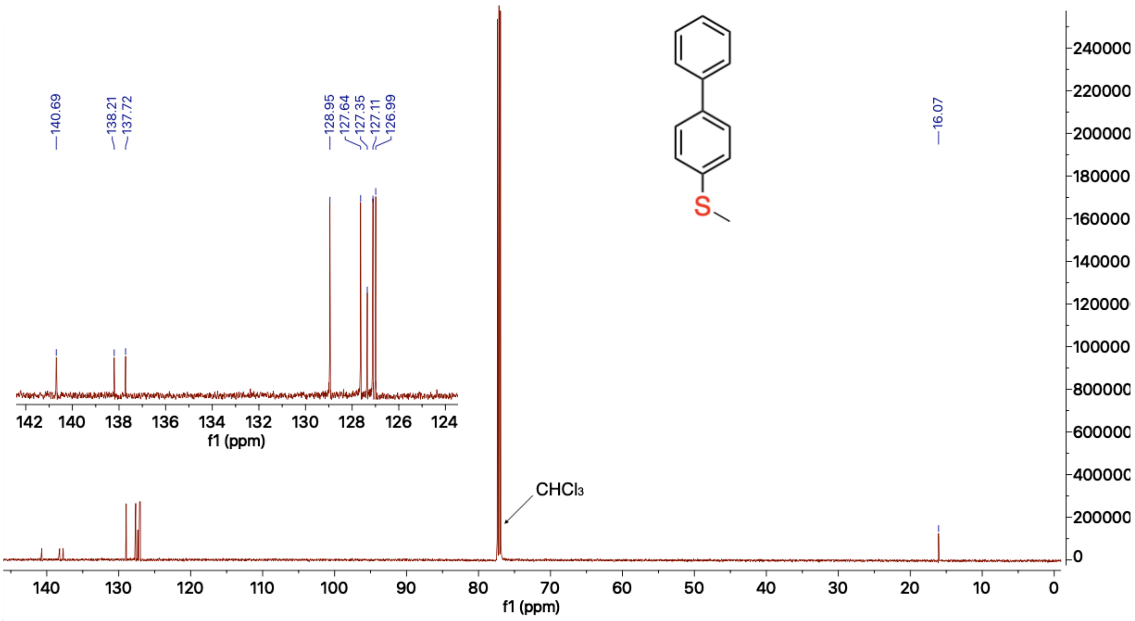

Figure S 2. ^{13}C NMR of **Ph$_2$-SMe** (151 MHz, $CDCl_3$).

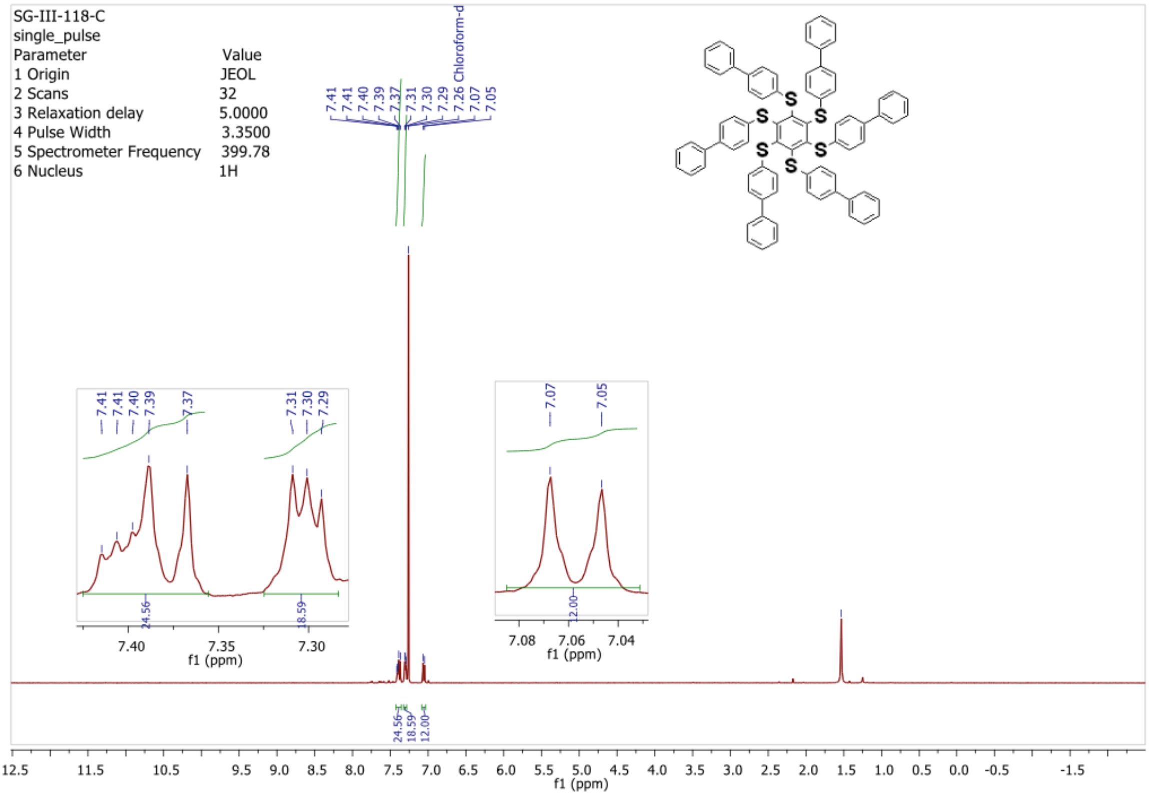

Figure S 3. ^{1}H NMR of **A6-Ph$_2$** (400 MHz, $CDCl_3$).

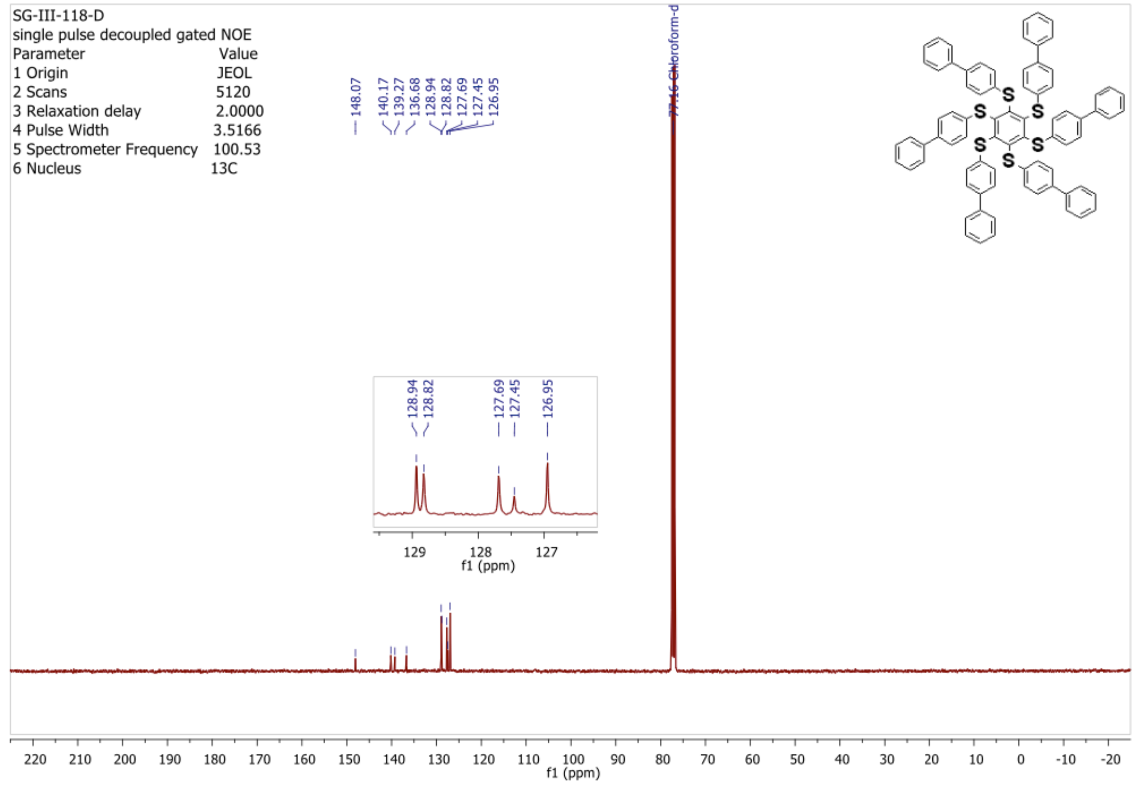

Figure S 4. ^{13}C NMR of **A6-Ph$_2$** (101 MHz, $CDCl_3$).

HRMS ANALYSES

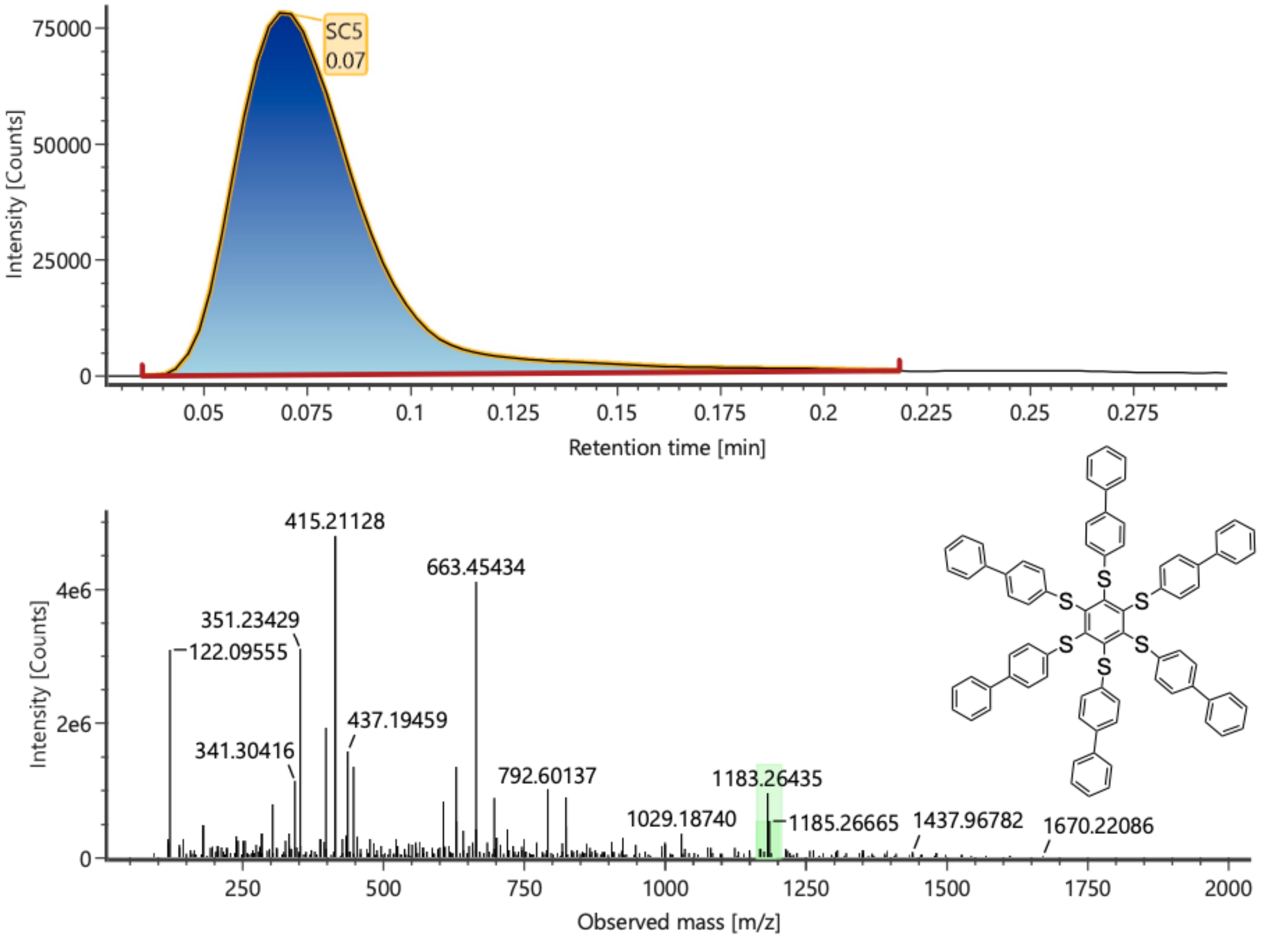

Figure S 5. HRMS analysis for **A6-Ph$_2$.**

XRD ANALYSES

General information

Single-crystal data for compounds **A6-Ph$_2$·DMF** and **A6-Ph$_2$·DMA** were collected at RT, on an Oxford XCalibur S CCD diffractometer equipped with a graphite monochromator (Mo-Kα radiation, λ = 0.71073Å). The structures were solved with SHELXT[6] by intrinsic phasing and refined on F^2 with SHELXL[7] implemented in the Olex[2] software[8] by full-matrix least squares refinement. All samples displayed crystal twinning. For **A6-Ph$_2$·DMA**, data were treated with the default configuration for twinned crystals of CrysAlisPro software, and structure solution and refinement were performed on the HKLF4 and HKLF5 files, respectively, containing the non-overlapped reflections; due to the strong overlapping, **A6-Ph$_2$·DMF** was instead treated as a single crystal. H_{CH} atoms for all compounds were added in calculated positions and refined riding on their respective carbon atoms. All non-hydrogen atoms were anisotropically refined and the rigid-body RIGU restraints applied,[7] except the disordered solvent molecules found within the crystal lattice, whose atoms were refined isotropically. See Table S1 for crystallographic details.

For the structural solution of **A6-Ph$_2$**, diffractograms in the 2θ range 3−70° (step size, 0.026°; time/step, 200 s; 0.02 rad soller; V × A / 40 × 40) were collected on a Panalytical X'Pert PRO automated diffractometer equipped with a PIXcel detector and operated in transmission geometry (capillary spinner), using Cu–Kα radiation without monochromator in the 2θ range 3° - 70° (continuous scan mode, step size 0.0260°, counting time 889.70 s, Soller slit 0.02, antiscatter slit ¼, divergence slit ¼, 40 mA × 40 kV). Five diffraction patterns were recorded and summed to enhance the signal to noise ratio. Powder diffraction data were analyzed with the software EXPO2014,[9] which is designed to analyze both monochromatic and non-monochromatic data. Selected peaks were chosen in the 2θ range 10–50°, and a unit cell of ca 2800 Å3 and with the most plausible space group $P2_1/c$ was found using the algorithm N-TREOR09,[10] consistent with an asymmetric unit comprised by half A6-Ph$_2$ molecule. Molecular fragment (asymmetric unit) was prepared from the molecular editor in EXPO2014[9] on the basis of the formerly determined crystal structure, and its molecular geometries optimized first with the molecular mechanics module (MM) by using the Universal Force Field (UFF),[11] and then with the semiempirical method MOPac (PV7),[12] both implemented in EXPO2014.[9] Structures were solved with the simulated annealing method without H-atoms (to reduce the degree of freedom). To the so-obtained structural solution, H-atoms were added in a calculated and the structural model optimized again with MM and MOPac-PV7 before the final Rietveld refinement. An overall thermal parameter for all the atoms was adopted. See Figure S6 for the pattern difference plot and Table S1 for crystallographic details. Crystal data can be obtained free of charge via www.ccdc.cam.ac.uk/conts/retrieving.html (or e-mail: deposit@ccdc.cam.ac.uk); CCDC numbers 2443649-2443651.

For phase identification purposes, X-ray powder diffractograms in the 2θ range 5-40° (step size, 0.02°; time/step, 20 s; 0.04 rad soller; 40mA x 40kV) were collected in Bragg-Brentano geometry, using Cu Kα radiation without a monochromator, on a Panalytical X'Pert PRO automated diffractometer equipped with an X'Celerator detector. The program Mercury[13] was used to calculate the X-ray powder patterns on the basis of single crystal data collected in this work. In all cases, the identity between polycrystalline samples and single crystals was always verified by comparing experimental and calculated powder diffraction patterns (See Figure S7).

Table S 1. Crystal data and refinement details for crystalline **A6-Ph$_2$**, **A6-Ph$_2$·DMF**, and **A6-Ph$_2$·DMA**.

	**A6-Ph$_2$**	**A6-Ph$_2$·DMF**	**A6-Ph$_2$·DMA**
**Formula**	$C_{78}H_{54}S_6$	$C_{85.5}H_{70}N_{2.5}O_{2.5}S_6$	$C_{88}H_{76.5}N_{2.5}O_{2.5}S_6$
**FW**	1183.65	1364.80	1401.37
**Temperature (K)**	300	295	295
**Cryst. System**	Monoclinic	Triclinic	Triclinic
**Space group**	$P2_1/c$	P-1	P-1
**Z**	4	2	2
**a (Å)**	22.0527(18)	9.9083(8)	9.9224(11)
**b (Å)**	6.2687(4)	18.370(2)	18.781(3)
**c (Å)**	21.9917(13)	20.8077(15)	20.719(2)
**α (deg)**	90	77.511(8)	79.014(12)
**β (deg)**	108.793(5)	80.032(6)	80.040(9)
**γ (deg)**	90	78.324(8)	79.107(11)
**V (Å^3)**	2878.1(4)	3588.0(6)	3684.2(9)
**D_{calc} (g/cm^3)**	1.366	1.263	1.263
**μ (mm^{-1})**	0.255	0.242	0.238
**Measured reflections**	-	19654	21581
**Independent reflections**	-	11943	21581
**Largest difference between peak and hole (e/Å^3)**	-	0.27/-0.23	0.34/-0.29
**R_1[on F_0^2, I>2σ(I)]**	-	0.0699	0.0832
**wR_2 (all data)**	-	0.1365	0.2335
**R_{wp} (%)**	6.1	-	-

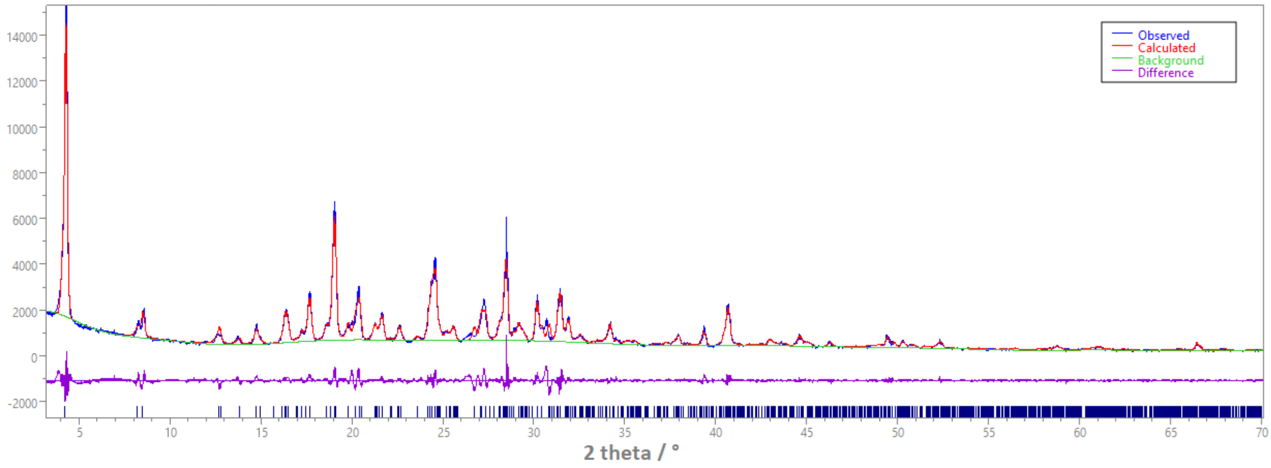

Figure S 6. Experimental (blue), calculated (red) powder XRD pattern by Rietveld refinement and difference profile (magenta) of **A6-Ph$_2$.**

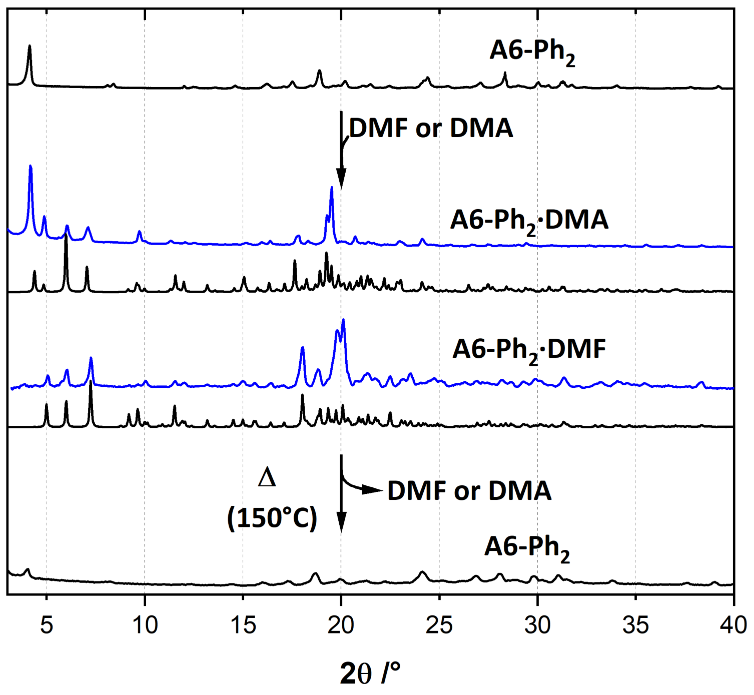

Figure S 7. Experimental recorded at RT (blue line) and calculated (black line) powder X-ray diffraction patterns for crystalline **A6-Ph$_2$·DMF** and **A6-Ph$_2$·DMA**.

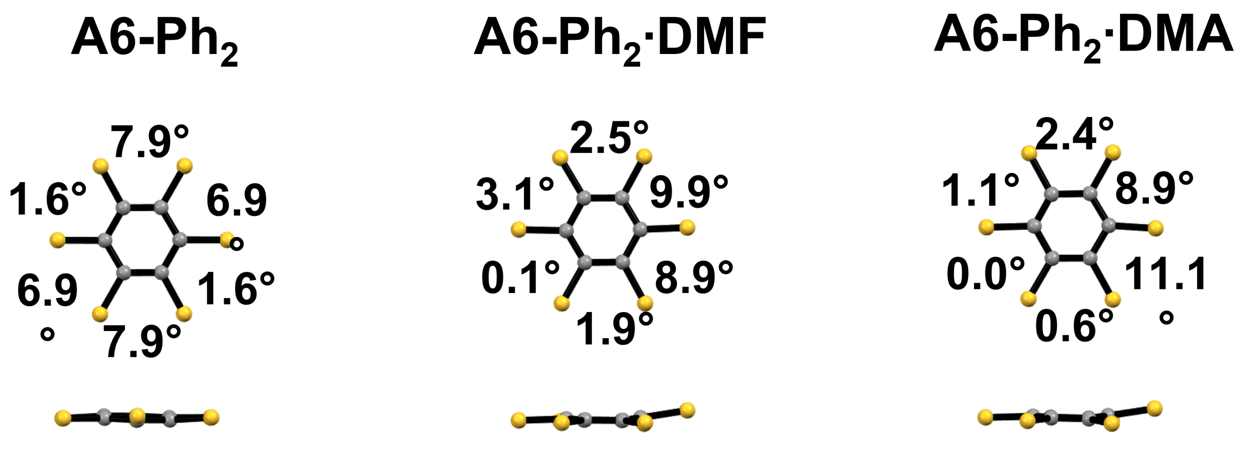

Figure S 8. Torsional angles in degrees between SC-CS bonds (top) and side views showing the distortions of the persulfurated benzenes cores (bottom) detected in crystalline **A6-Ph$_2$, A6-Ph$_2$·DMF** and **A6-Ph$_2$·DMA.**

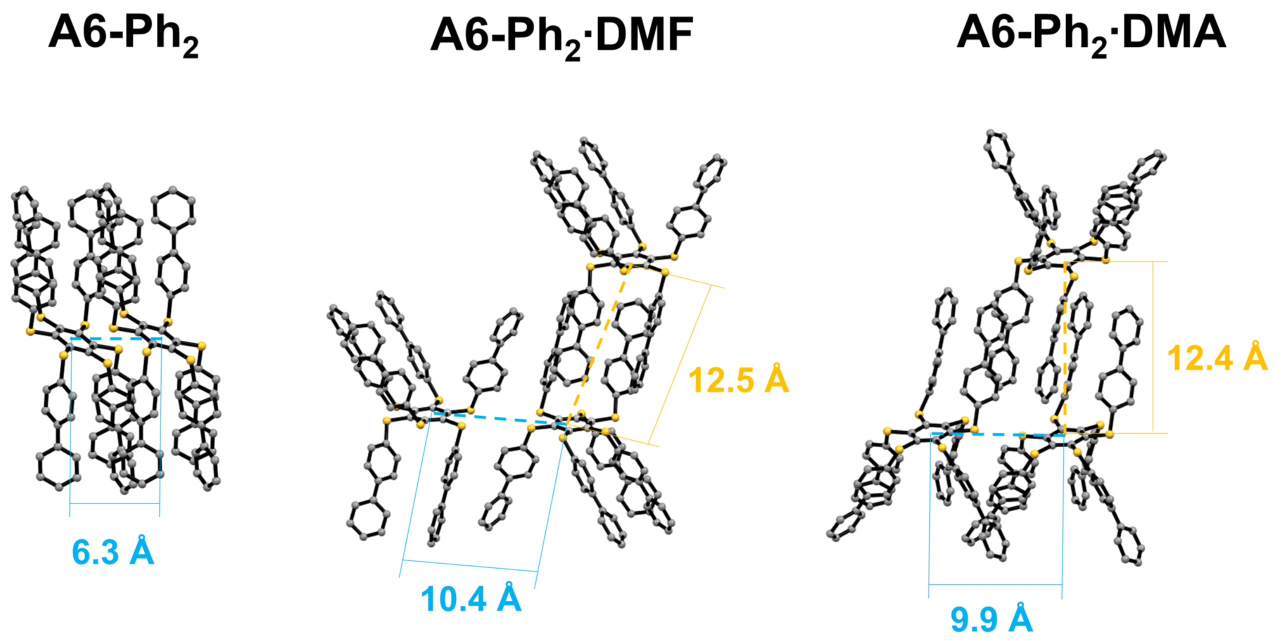

Figure S 9. Centroid-to-centroid distances between the persulforated benzene cores detected in crystalline **A6-Ph$_2$**, **A6-Ph$_2$·DMF** and **A6-Ph$_2$·DMA**. H_{CH} atoms, DMF and DMA molecules are not shown for clarity.

TGA ANALYSES

General information

TGA measurements were performed with a PerkinElmer TGA800 instrument in the temperature range 40–500°C under a flow of N_2 gas at a heating rate of 10.00 °C•min^{-1}.

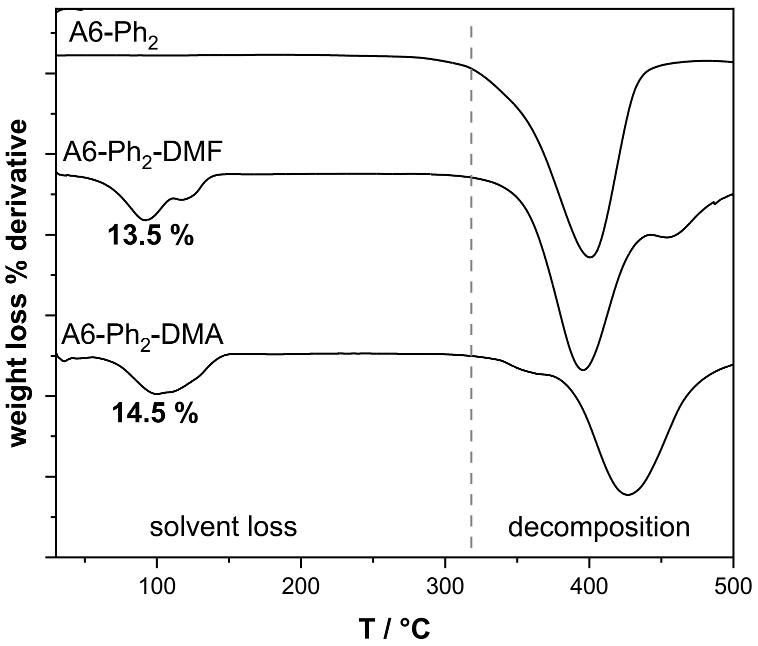

Figure S 10. Thermogravimetric analyses recorded on polycrystalline samples of **A6-Ph₂, A6-Ph₂·DMF** and **A6-Ph₂·DMA.**

PHOTOPHYSICAL ANALYSES

General information

All photophysical analyses were carried out in CH_2Cl_2 at 298 K, unless otherwise specified. UV–vis absorption spectra were recorded with a PerkinElmer λ40 spectrophotometer using quartz cells with path length of 1.0 cm. The UV–vis diffuse reflectance spectra were recorded on a Perkin-Elmer 365+ spectrophotometer fitted with a 50mm integrating sphere ($BaSO_4$ coating). Luminescence spectra and lifetimes were recorded and detemined with a PerkinElmer LS-50, an Edinburgh FS5 or an Edinburgh FLS920 spectrofluorometer (equipped with a Hamamatsu Photomultiplier R928P phototube) or on a Varian Cary Eclipse phosphorimeter. Lifetimes shorter than 10 μs were measured by the same Edinburgh FLS920 spectrofluorometer by time-correlated single-photon counting (TCSPC) technique. Quantum yields of solutions at RT where determined following the method of Demas and Crosby,[14] using naphthalene or quinine sulfate as standards ($\Phi_{fl.}$ = 0.23 in air equilibrated cyclohexane and $\Phi_{fl.}$ = 0.55 in 0.5 M H_2SO_4, respectively). Quantum yields of solid samples are determined by means of an integrating sphere (LabSphere, 4 in. diameter) with the method developed by De Mello.[15] Quantum yields of rigid matrices at 77 K were calculated with the same apparatus using an homemade optical glass dewar flask, fitting the above-mentioned integrating sphere vertically. The estimated experimental errors are 2 nm on the band maximum, 5% on the molar absorption coefficient and luminescence lifetime and 20% on emission quantum yields. Measurements of emission spectra and lifetimes as a function of temperature were performed by inserting a glass vessel containing ca. 50 mg of solid sample into a quartz cell, the latter fitted into a cryostat by Oxford Instruments, loaded with liquid N_2 and equipped with a temperature controller (model ITC1).

Vapochromism experiments. Samples used for tests on the vapochromic behaviour of solid **A6-Ph₂** at RT were prepared as reported in Scheme S1, using DMF, C_6H_6, $CHCl_3$ or CH_2Cl_2. No change in the emission has been observed in the presence of CH_2Cl_2, $CHCl_3$ or benzene employing the same experimental conditions. Micrographs of solid samples were taken with a Cainda digital camera (40x-1000x) on a metal stand, connected to a computer. Photographs on macroscopic samples were taken with the digital camera of a smartphone (model: Apple iPhone 13 mini; operating system: iOS 18.5).

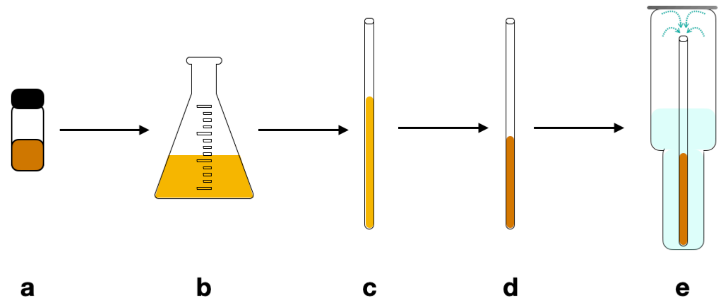

Scheme S 1. Preparation of samples for analysis of the luminescence in vapochromism tests: a) isolated solid material; b) dissolution in toluene; c) transfer of the solution in a glass tube; d) slow evaporation of the solution leads to the coating of the glass tube with the solvent-free solid material; e) transfer of the tube into a sealed glass vessel containing DMF allows the contact between vapours of the solvent and the solvent-free solid material. The vessel can be fitted into a spectrofluorometer sample-holder for analysis.

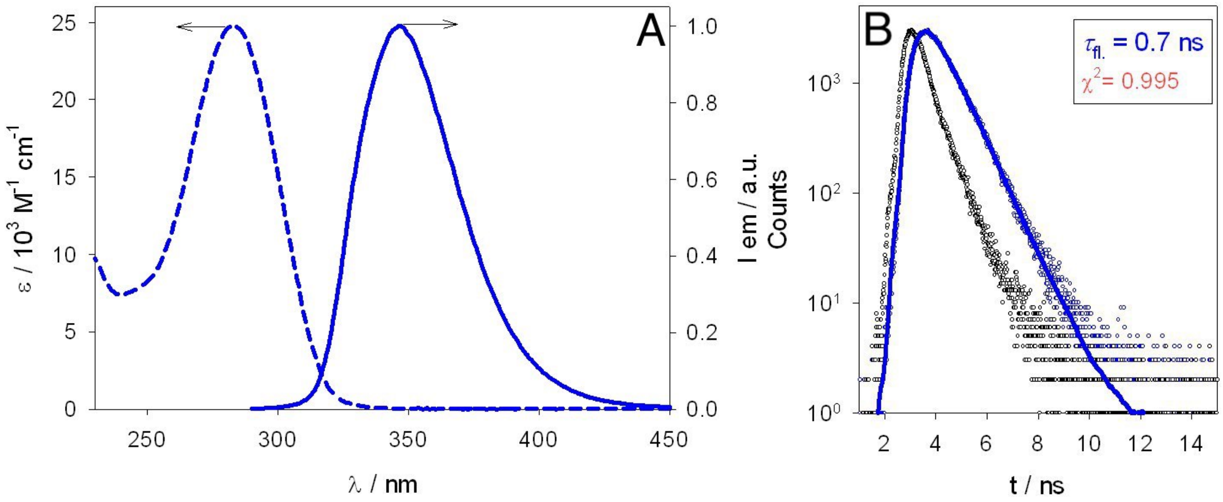

Figure S 11. A: absorption (dashed) and normalized emission spectra of **Ph_2-SMe** in air-equilibrated CH_2Cl_2 at RT (λ_{ex} = 270 nm; λ_{max} = 347 nm; $\Phi_{fl.}$ = 0.89). B: emission decay recorded in the same experimental conditions (blue dots) and corresponding monoexponential fitting curve (solid line). λ_{ex} = 270 nm, λ_{em} = 350 nm; the instrumental response function (IRF) is also shown (black dots).

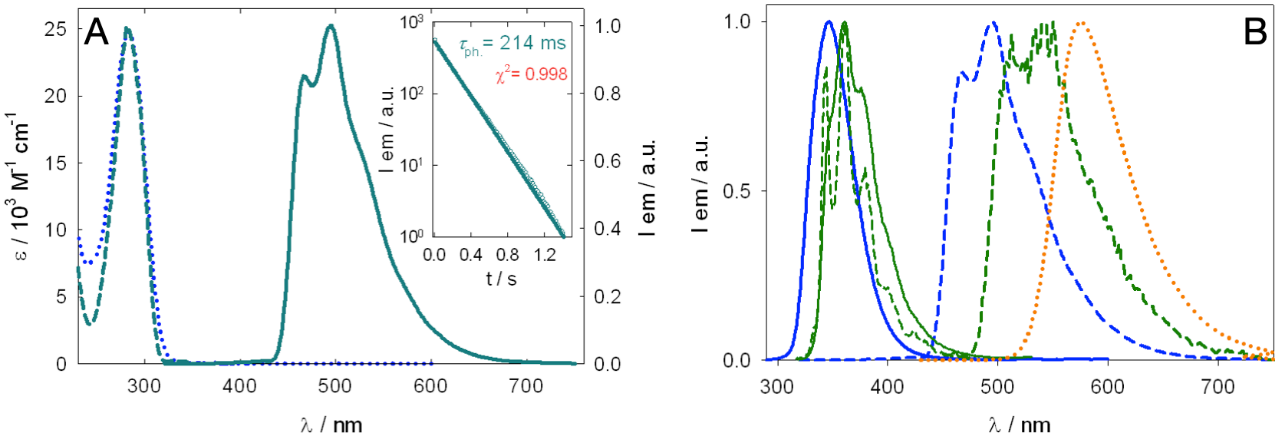

Figure S 12. A: comparison between phosphorescence spectrum of **Ph_2-SMe** (solid line, λ_{ex} = 290 nm; delay time = 1 ms; gate time 200 ms), its corresponding excitation spectrum (dashed line; λ_{ex} = 480 nm) collected at 77 K in CH_2Cl_2:CH_3OH (1:1, v/v) and the absorption spectrum in CH_2Cl_2 solution at RT (dotted line). Inset: phosphorescence lifetime detected at λ_{em} = 470 nm (gate time = 15 ms) at 77 K and corresponding monoexponential fitting function (solid line). B: comparison of emission profiles of **Ph_2-SMe** in solution (blue: full line fluorescence at RT, dashed line phosphorescence at 77 K) and from powders (green: full line fluorescence at RT; short dashed line fluorescence at 77 K; long dashed line phosphorescence at 77K). The emission of solid **A6-Ph_2** at RT is also shown for comparison (dotted orange line).

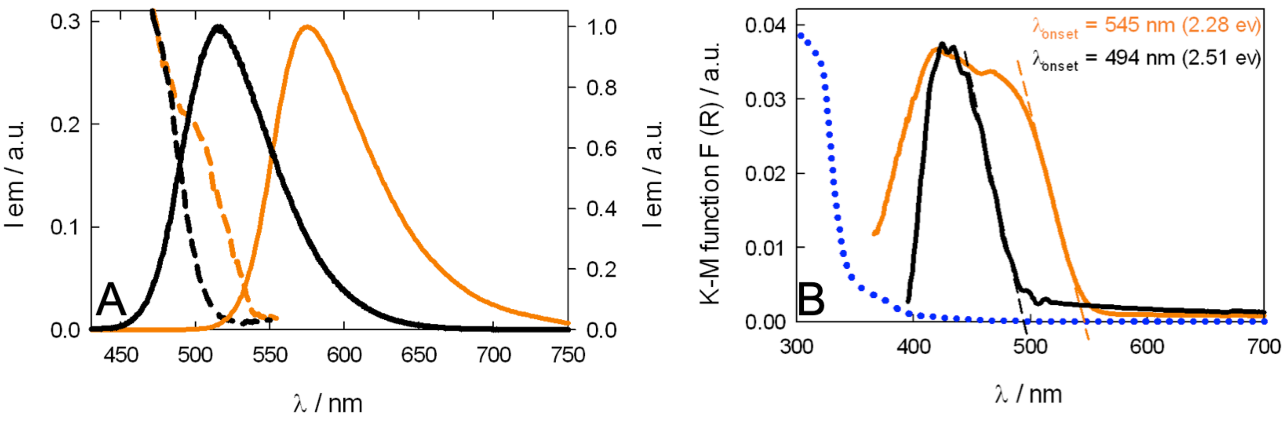

Figure S 13. A: comparison between emission spectra recorded at RT for solid **A6-Tol** and **A6-Ph_2** (black and orange lines, respectively; 350 < λ_{ex} < 390 nm) and their corresponding excitation spectra (λ_{em} = 570 nm and 580 nm, respectively). B: Kubelka-Munk function (F(R)) calculated for solid **A6-Tol** and **A6-Ph_2** (black and orange lines, respectively) at RT from diffuse reflectance spectra, according to $F(R)=(1-R)^2/2R$. The trace of solid **Ph_2-SMe** is also shown (blue dotted line).

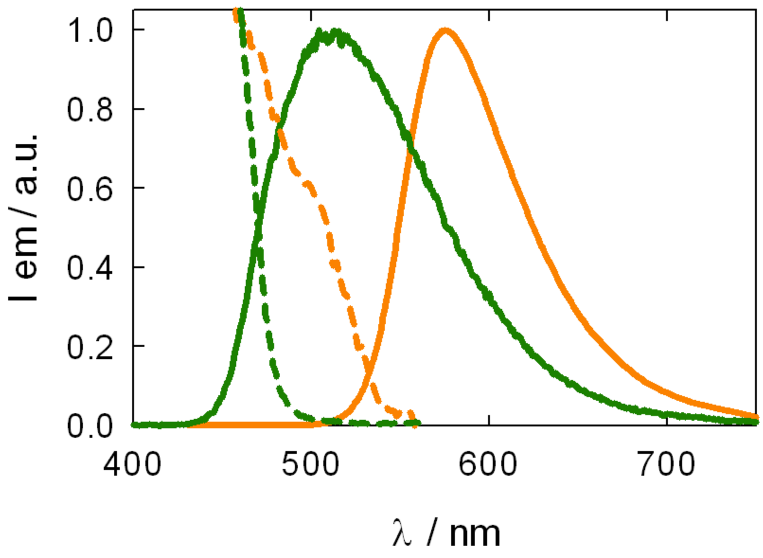

Figure S 14. Comparison between emission (solid lines) and excitation spectra (dashed lines) recorded at RT from solid samples of solvent-free **A6-Ph₂** (orange) and its solvated form **A6-Ph₂•DMF** (green). λ_{ex} = 350 nm; λ_{em} = 580 nm.

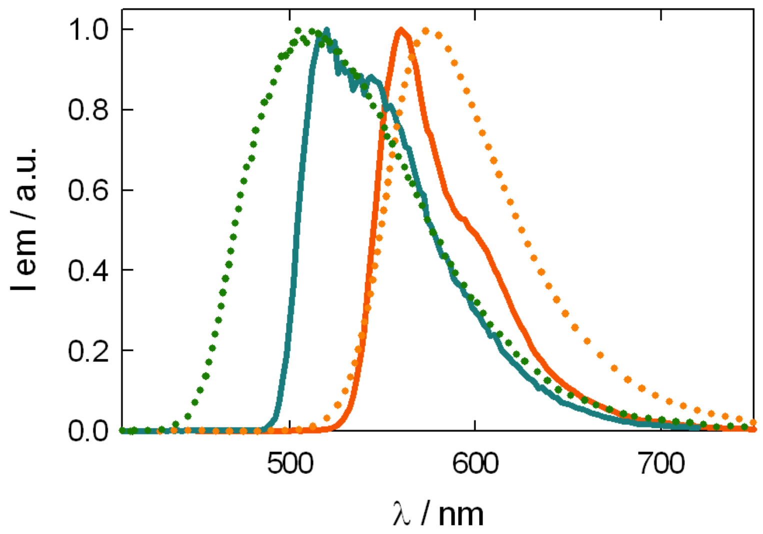

Figure S 15. Comparison between emission spectra recorded at 77 K (solid lines) and at RT (dotted lines) from solid samples of solvent-free **A6-Ph₂** (orange) and its solvated form **A6-Ph₂•DMF** (green). λ_{ex} = 350 nm.

Table S 2. Emission data for crystalline phases at 77 K.

Compound	λ_{max} (nm)	τ (ms)
**A6-Tol** [a]	495	0.095
**A6-Ph$_2$**	555	0.3 - 1.1 [b]
**A6-Ph$_2$•DMF**	520	1.2 - 4.6 [b]

[a] Data from ref. [16].
[b] Biexponential fitting.

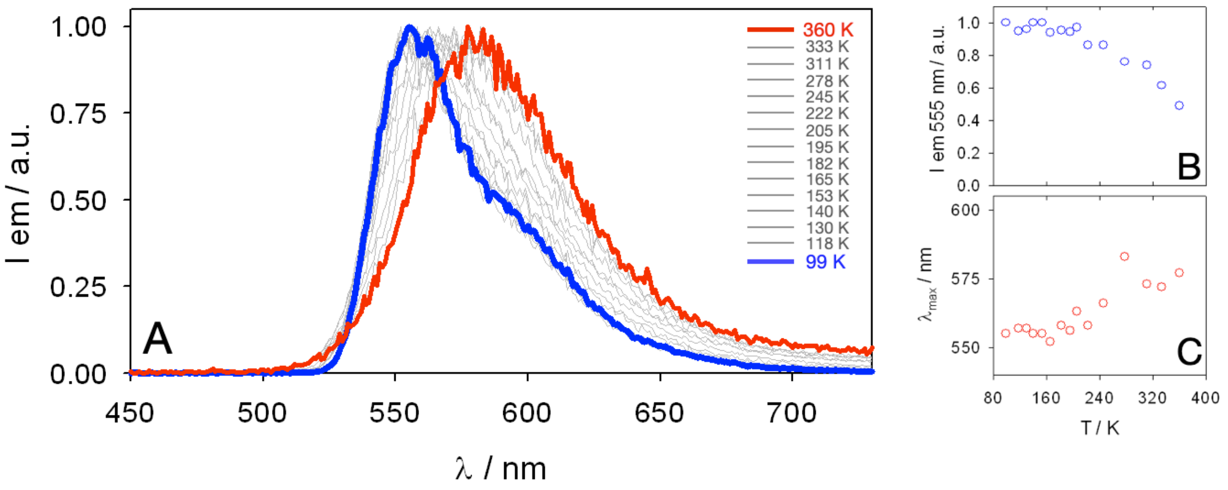

Figure S 16. A: temperature-dependent emission spectra of crystalline **A6-Ph$_2$** between 99 (blue line) and 360 K (red). B-C: associated changes in the emission intensity at 555 nm and in the emission maxima as a function of temperature, respectively. λ_{ex} = 380 nm; delay time: 0.1 ms; gate time: 10 ms.

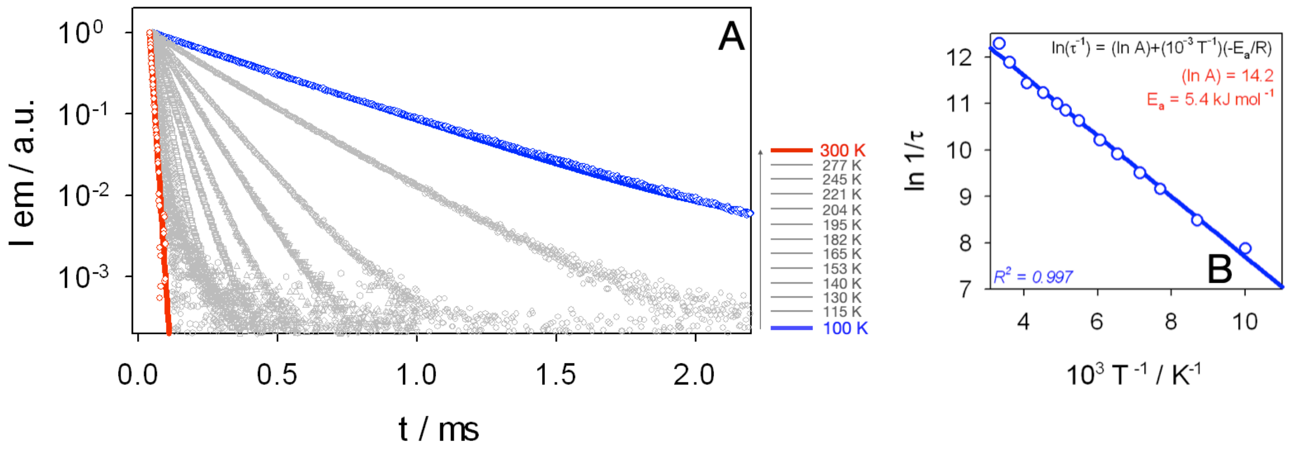

Figure S 17. A: temperature-dependent emission lifetimes of crystalline **A6-Ph$_2$** between 100 (blue dots) and 300 K (red dots), with corresponding monoexponential fitting functions (solid lines). λ_{ex} = 380 nm; λ_{ex} = 560 nm; delay time: 0.05 ms. B: associated Arrhenius plot obtained by the dependence of [ln(τ^{-1})] vs. 10^3T^{-1}.

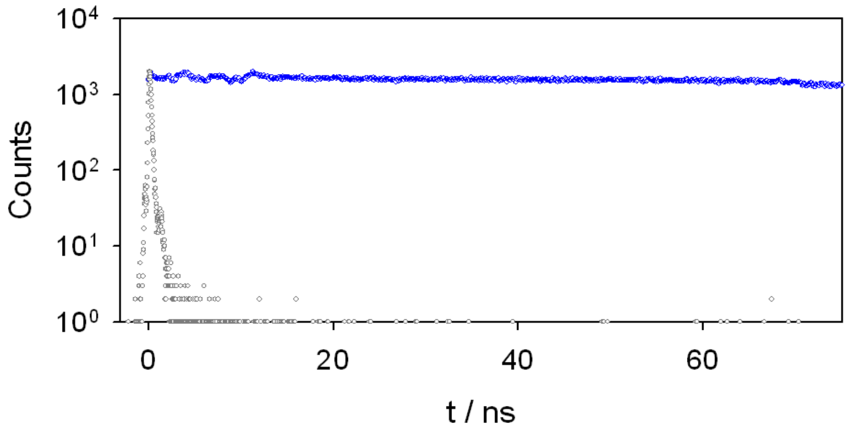

Figure S 18. Emission decay in the ns range for solid **A6-Ph$_2$** at RT (λ_{em} = 570 nm; blue dots); the instrument response function (IRF; λ_{ex} = 365 nm) is also plotted as the grey dots. No fitting exponential function is shown (not relevant in this time range).

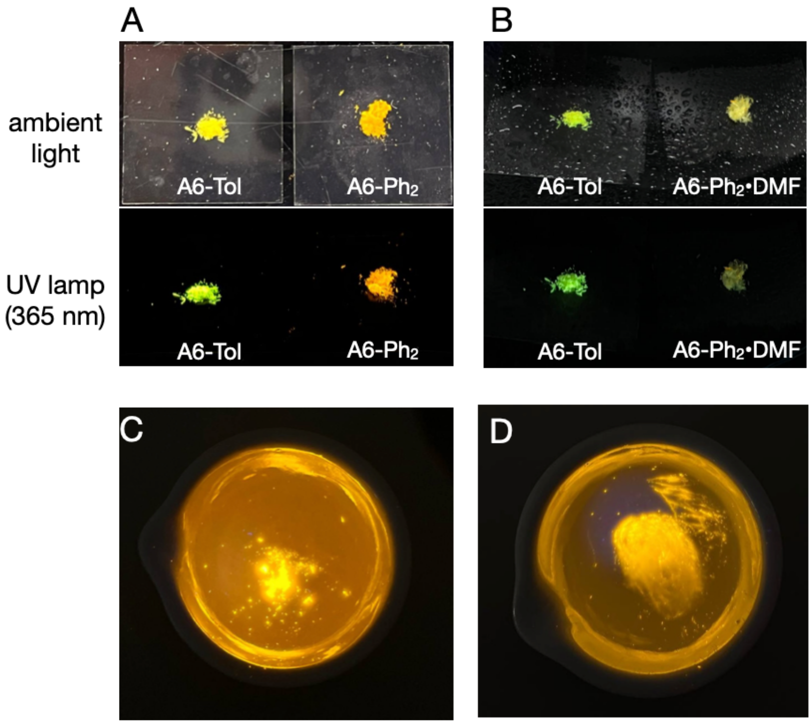

Figure S 19. Photographs of samples of crystalline **A6-Tol** and **A6-Ph$_2$** at RT before (A) and after (B) exposure to DMF vapours for 2 days. Photographs in the upper panel are taken under ambient light, while under UV excitation at the bottom. No appreciable change is observed for model compound **A6-Tol**. A qualitative test on the luminescence properties of **A6-Ph$_2$** at RT before (C) and after (D) grinding in a mortar is also shown (λ_{ex} = 365 nm). Small differences in the displayed color depend on the automatic white balance of the camera employed.

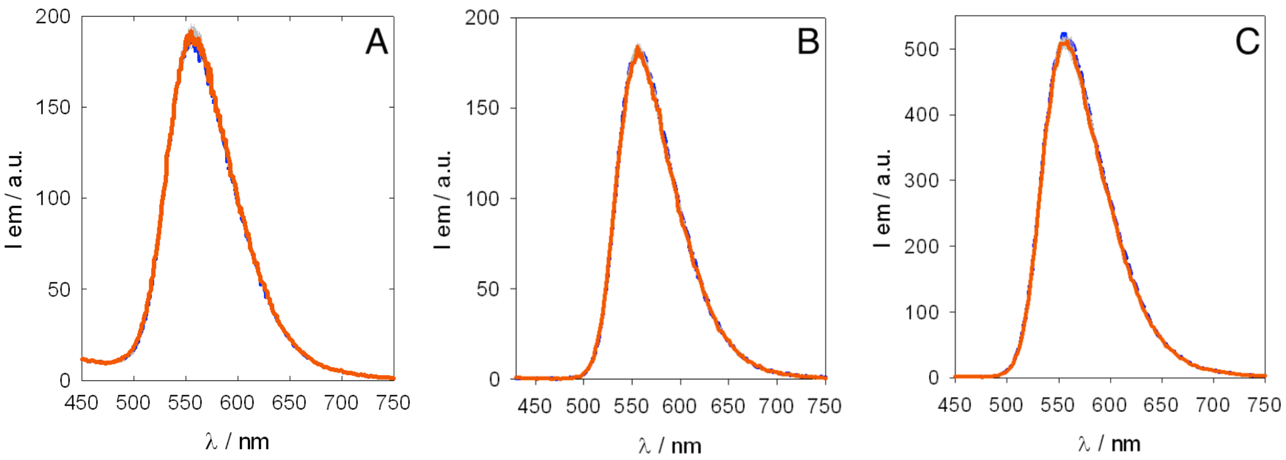

Figure S 20. Emission changes in solid **A6-Ph$_2$** upon exposure to solvent vapours (A: C_6H_6; B: $CHCl_3$; C: CH_2Cl_2) at RT for 24h (1 acquisition per hour). λ_{ex} = 350-355 nm.

COMPUTATIONAL DETAILS

Quantum-chemical (QC) calculations were carried out on the *aaabbb* and *ababbb* conformers of isolated **A6-Ph$_2$** and on model aggregates (dimers and trimers). For the *ababbb* conformer the same calculations carried out for the isolated molecule were repeated including two explicit DMF solvent molecules. In addition, ground and excited states of the model system **Ph$_2$-SMe** were also investigated. The optimized ground state geometries of **Ph$_2$-SMe** and **A6-Ph$_2$** were determined at M06-2X/def2-SVP level of theory including the D3 version of Grimme's dispersion.[17] Vertical excitations energies were evaluated at TD-M06-2X level with the same basis set and D3 dispersion. Excited state geometries were obtained at TD-M06-2X-D3/def2-SVP level or with UM06-2X-D3/def2-SVP. In addition, calculations using the CAM-B3LYP functional were also carried out to confirm the nature of low-lying excited states, although the M06-2X functional was considered for comparison with experimental data due to its closer agreement.
To assess the role of excitonic interactions, excited state calculations at TD-M06-2X/def2-SVP+D3 level were carried out on model aggregates (dimers and trimers) of **A6-Ph$_2$**. At the ground state optimized geometry of the *aaabbb* and *ababbb* conformers, we also computed the excitation energies of the central molecule surrounded by the crystal/amorphous environment, with QM/MM calculations. QM/MM calculations were performed with the Gaussian16 package[18] and the ONIOM model,[19] using the M06-2X functional and def2-SVP basis set for the high-level region (i.e. a central **A6-Ph$_2$** molecule) and the QM calculations included electronic embedding. The low-level region (molecular mechanics) was modelled by atomic point charges determined by the charge equilibration (Qeq) approach[20] using the Dreiding force field[21] parameters attributed to the fixed molecular geometry of the surrounding **A6-Ph$_2$** molecules. Interestingly, in contrast to *aaabbb* embedded crystal, the energy difference between T(nπ*/ $\pi\pi$*) and T(Ph$_2$*) states for *ababbb* conformer is little affected by the environment and remains almost identical (Table S7), an indication that intermolecular interactions (larger in the *aaabbb* crystal compared to the solvated *ababbb* crystal) are responsible for tuning excited state energies.
All the QC calculations were carried out with the Gaussian16 package.[18]

At the optimized ground-state geometry, both conformers of **A6-Ph$_2$** exhibit a similar pattern for the lowest-lying triplet states compared to parent **A6-Tol** (see Tables S3 and S4). The lowest triplet state of **A6-Ph$_2$** *aaabbb* has a dominant nπ* nature, while **A6-Ph$_2$** *ababbb* is predominantly $\pi\pi$*. As in **A6-Tol**, the $\pi\pi$* nature induces significant excited-state distortion, reducing the S_0/T_1 energy gap and promoting non-radiative deactivation – explaining the lower RT luminescence quantum yield of the solvated crystal phase, in which the *ababbb* conformation is observed. Above these states, a third triplet state with a localized excitation on **Ph$_2$** arms (indicated as Ph$_2$*) emerges.
Triplet-state geometry optimization predicts an **A6-Ph$_2$** *aaabbb* emission energy from the nπ* triplet of 2.30 eV, close to that of **A6-Tol** *aaabbb* conformer (2.32 eV, see Table S5, SI), conflicting with experimental data that indicate a remarkable red-shift compared to the solid-state emission of the latter. Although enhanced excitonic interactions could be considered as a possible explanation for the observed red shift, dimer and trimer aggregate calculations on the *aaabbb* conformer do not evidence this expectation. However, calculations on the aggregate reveal a slight increase in the excitation energy of the lowest nπ* triplet state, while the Ph$_2$* states exhibit a downward energy shift (see Table S6, SI). This suggests that aggregation lowers the Ph$_2$* state in *aaabbb* conformers. QM/MM calculations further support this result, showing a stronger stabilization of Ph$_2$* triplet states in the solvent-free crystal (*aaabbb* conformers) than in the solvated crystal (*ababbb* conformation), as a result of the closer packing of **A6-Ph$_2$** molecules in the former (see Table S7, SI).
The emission energy computed for the Ph$_2$* excited state of isolated **A6-Ph$_2$** *aaabbb* (2.26 eV, Table S5) can be compared with that of the model system **Ph$_2$-SMe** (2.36 eV, Table S5). Although the two emissions were computed for isolated molecules, while experimental data were measured in the solid phase, their difference (2.36 vs. 2.26 eV) aligns well with the difference between the experimental emission energies (ca. 2.30 eV for **Ph$_2$-SMe**, see Figure S12B, vs. 2.23 eV for **A6-Ph$_2$** *aaabbb*, see Table S2). This similar trend adds support to the assignment of phosphorescence from the Ph$_2$* state for **A6-Ph$_2$** *aaabbb*.

The consistency of computed emission energies for the Ph_2* triplet state of both **Ph_2-SMe** and **A6-Ph_2** in *aaabbb* conformation (see Table S5), along with intermolecular interactions that strongly indicate stabilization of the Ph_2* triplet state, suggest that an inversion of the nπ* and Ph_2* triplet state minima is likely to occur in the solid of **A6-Ph_2** *aaabbb*, as illustrated in Figure 3 (main text). In contrast, for **A6-Ph_2** in *ababbb* conformation, the minimum of the Ph_2* triplet state remains 9.4 kcal/mol higher than that of the lowest ππ* triplet state for isolated molecule calculations. This energy difference increases to 12.2 kcal/mol when two DMF solvent molecules are explicitly included in the calculations, confirming that emission originates from the ππ* state. Intermolecular constraints in the solid phase likely reduce geometric distortion compared to isolated molecule calculations (Figure S26, SI), resulting in an emission energy consistent with experimental observations.

Molecular dynamics (MD) simulations were carried out on a 5×5×5 supercell of **A6-Ph_2** molecules. The aim was to explore the possibility of isomerization from conformer *aaabab* to *aaabbb* observed experimentally. Thus, the crystal structure of **A6-Ph_2•DMF** was considered. Because isomerization occurs, experimentally, upon evaporation of the solvent, to facilitate isomerization during the MD simulations, these were carried out in the NVT ensemble, starting from the **A6-Ph_2•DMF** structure with solvent molecules removed. Both the MM3[22] and recently-improved OPLS all-atom force field[23] (FF) were used with Tinker[24] and LAMMPS[25] software packages, respectively. The MM3 simulation was 2 ns long and to speed-up isomerization and avoid unphysical aggregation observed at lower temperatures, the temperature was set to 800K. The OPLS simulations, were performed at different temperatures (from 300K to 800K) using standard and adapted force field parameters. Specifically, both ChelpG charges and the newly developed Density Derived Electrostatic and Chemical (DDEC6) net atomic charges were considered, the latter chosen due to their better description for different conformers.[26] The ChelpG charges were used along with the original VdW interaction parameters as described by Jorgensen et al.[23] The DDEC6 charges, determined using the *chargemol* program (https://sourceforge.net/projects/ddec/), were used to parametrize VdW parameters according to the Tkatchenko-Sheffler[27] method using a slightly modified script from ref[28]. Finally, for the CCSC torsional angle, the second Fourier coefficient V_2 describing the torsional potential energy[29] was set to 1.70 (to be in line with the MM3 force field. The total simulation time ranged from 10 to 20ns.
The changes in sign of the six CCSC dihedral angles were monitored during the MD simulations. Isomerization of selected molecules were detected both from MM3 and OPLS MD simulations. As an example, the sharp change of dihedral angle leading to isomerization is shown in Figure S32. Thus, the MD simulations showed that the change of conformer, starting from the original *ababbb* is possible. Thanks to the flexibility of these molecules, different conformers were generated.

Table S 3. Absolute energies (a.u.) of the optimized ground and triplet excited states ((nπ*/ ππ*) and (Ph_2*) nature) of **A6-Ph₂** conformers studied in this work along with the energy difference between these two triplet states at their respective optimized geometries. From M06-2X/def2-SVP and TD-M06-2X/def2-SVP calculations including D3 dispersion correction.

Conformer	S_0	T (nπ*/ππ*)	T (Ph_2*)	ΔE[T (Ph_2*)- T (nπ*/ ππ*)] / kcal/mol
**A6-Ph₂**-*aaabbb*	-5389.816800	-5389.720080	-5389.713782	3.95
**A6-Ph₂**-*ababbb*	-5389.806640	-5389.736415	-5389.721368	9.44
**A6-Ph₂**-*ababbb*+2DMF	-5886.272557	-5886.187757	-5886. 168334	12.19

Table S 4. Computed vertical excitation energies (eV) at the optimized ground state geometry of the two **A6-Ph₂** conformers. From TD-M06-2X/def2-SVP calculations supplemented with the D3 dispersion correction.

Conformer	T_1 / eV	T_2 / eV	T_3 / eV
**A6-Ph₂**-*aaabbb*	2.91 (nπ*) [a]	3.16 (nπ*/ππ*) [a]	3.44 (Ph_2*) [a]
**A6-Ph₂**-*ababbb*	3.11 (ππ*)	3.34 (ππ*/nπ*) [a]	3.41 (Ph_2*) [a]
**A6-Ph₂**-*ababbb*+2DMF	3.09 (ππ*)	3.34 (Ph_2*) [a]	3.35 (Ph_2*) [a]
**Ph₂SMe**	3.55 (Ph_2*) [a]	-	-

[a] The dominant character of the excited state.

Table S 5. Computed vertical emission energies (eV) at the optimized triplet state geometries ((nπ*/ππ*) and (Ph_2*) triplet states) of the two **A6-Ph₂** conformers. From TD-M06-2X/def2-SVP calculations in vacuo supplemented with the D3 dispersion correction.

Conformer	T_1(nπ*/ππ*)→ S_0 @ T_1(nπ*/ππ*) geometry / eV	T_1(Ph_2*)→ S_0 @ T_1(Ph_2*) geometry / eV	Exp
**A6-Tol**-*aaabbb*	2.32	-	2.39[a]
**A6-Ph₂**-*aaabbb*	2.30	2.26	2.23[b]
**A6-Ph₂**-*ababbb*	1.72	2.32	2.38[b]
**A6-Ph₂**-*ababbb*+2DMF	1.79	2.34	2.38[b]
**Ph₂SMe**	-	2.36	2.30/2.48[c]

[a] Data from ref. 16; [b] see Table S 2; [c] see Figure S 12.

Table S 6. Comparison of TD-M06-2X/def2-SVP computed vertical excitation energies (E / eV) for the lowest triplet excited states of a single **A6-Ph$_2$** molecule, *aaabbb* conformer, and for a dimer and trimer of the same molecule extracted from the crystal structure.

**Excited state**	**Monomer** E / eV	**Dimer** E / eV	**Trimer** E / eV
$\mathbf{T_1}$ (nπ*)	2.83	2.85	2.85
$\mathbf{T_2}$ (nπ*/ ππ*)	3.04	3.07	3.07
$\mathbf{T_3}$ (Ph_2*)	3.22	3.20	3.18

Table S 7. Comparison between TD-M06-2X/def2-SVP computed vertical excitation energies (E / eV) of the lowest triplet excited states of an **A6-Ph$_2$** molecule, *aaabbb* and *ababbb* conformer in its crystal geometry, embedded in the crystal environment (from QM-MM calculations) or in gas phase.

**Excited state**	**Gas phase** E / eV	**Gas phase** $\Delta E(T_3 - T_n)$/eV	**Crystal embedding** E / eV	**Crystal embedding** $\Delta E(T_3 - T_n)$/eV
	**A6-Ph$_2$**-*aaabbb*			
$\mathbf{T_1}$ (nπ*/ ππ*)	2.83	0.39	2.88	0.32
$\mathbf{T_2}$ (nπ*/ ππ*)	3.04	0.18	3.10	0.10
$\mathbf{T_3}$ (Ph_2*)	3.22	0.00	3.20	0.00
	**A6-Ph$_2$**-*ababbb*			
$\mathbf{T_1}$ (nπ*/ ππ*)	3.12	0.27	3.13	0.26
$\mathbf{T_2}$ (nπ*/ ππ*)	3.28	0.11	3.29	0.10
$\mathbf{T_3}$ (Ph_2*)	3.39	0.00	3.39	0.00

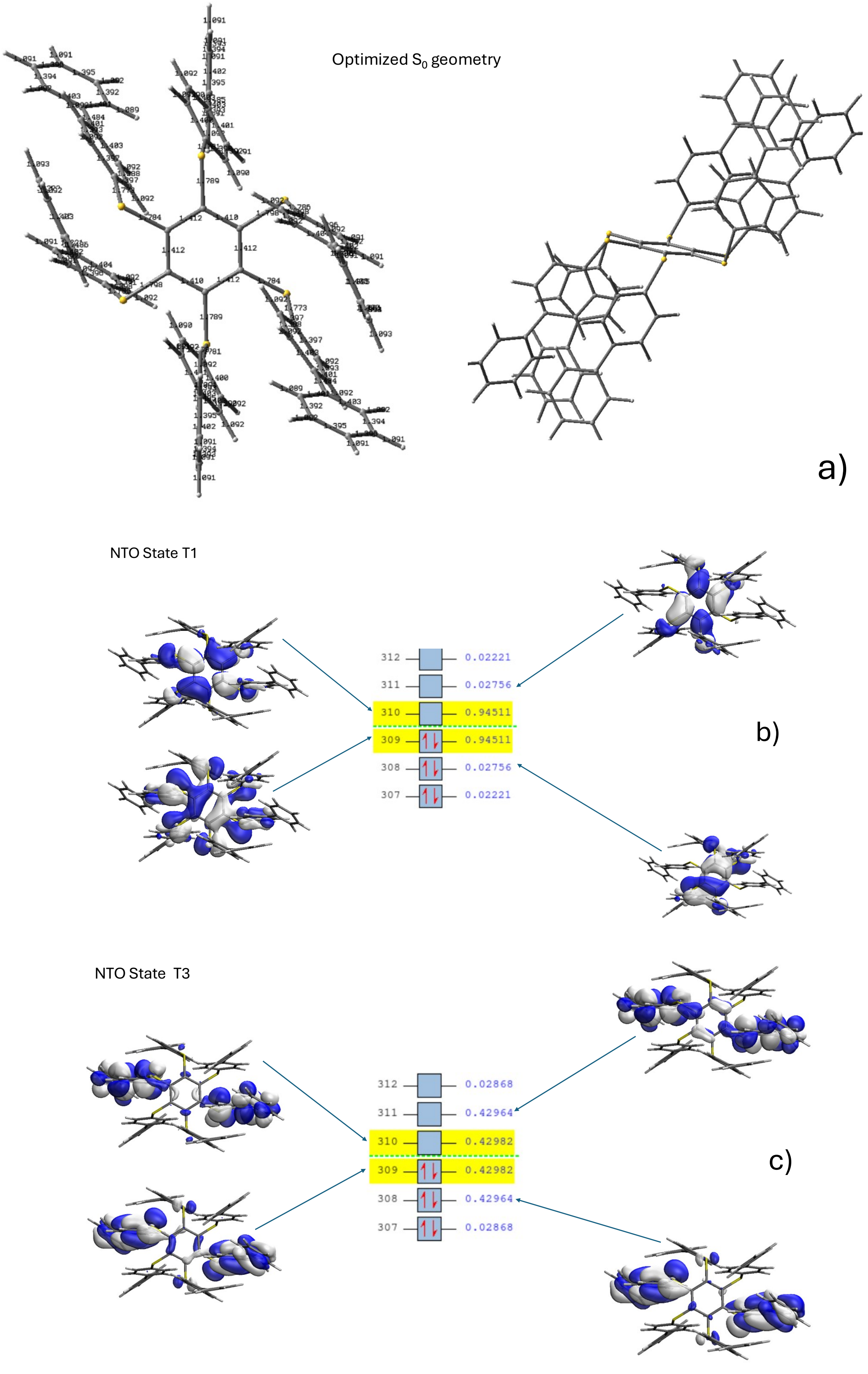

Figure S 21. A6-Ph₂, *aaabbb* conformer: a) Optimized ground state geometry (M06-2X/def2-SVP), b) NTO analysis of the lowest triplet excited state showing dominant nπ* character (TD-M06-2X/def2-SVP, see Table S4), c) NTO analysis of the third triplet state showing with Ph_2* character (TD-M06-2X/def2-SVP, see Table S4).

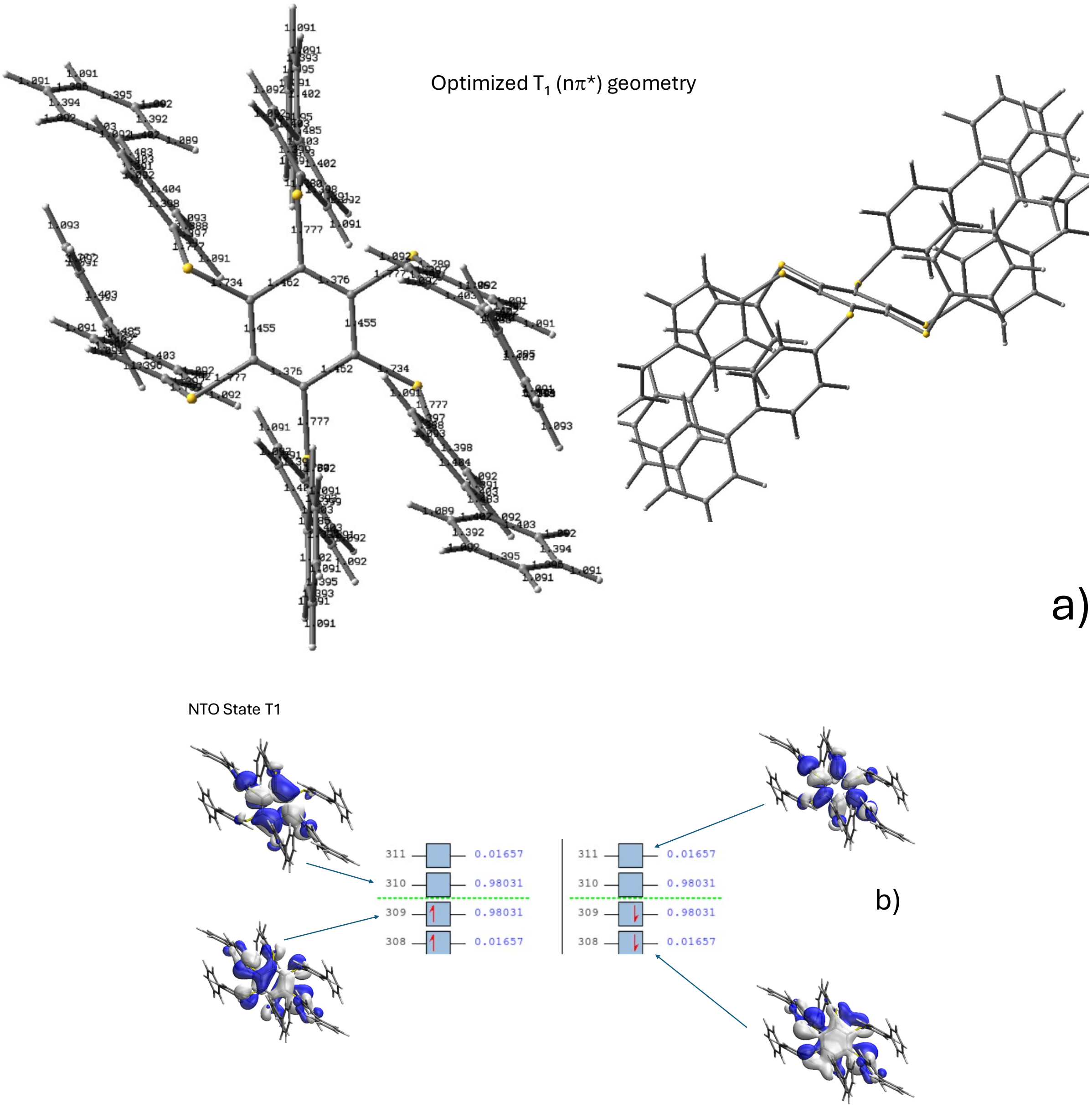

Figure S 22. A6-Ph₂, *aaabbb* conformer: a) Optimized geometry of the lowest triplet state showing dominant nπ* character (TD-M06-2X/def2-SVP), b) NTO analysis of the excited state wavefunction showing dominant nπ* character (TD-M06-2X/def2-SVP).

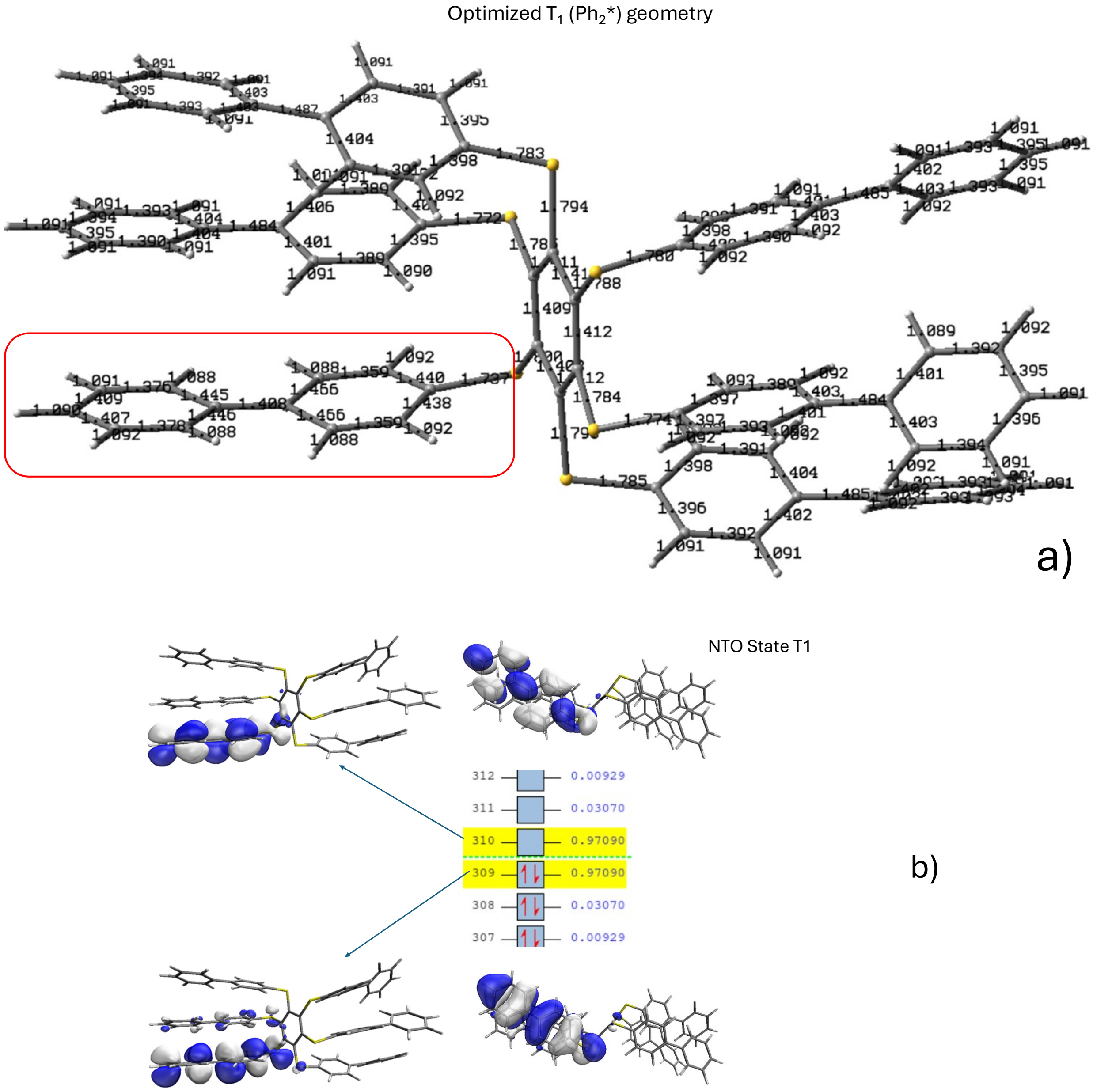

Figure S 23. A6-Ph₂, *aaabbb* conformer: a) Optimized geometry of the lowest triplet state showing dominant Ph_2* character (TD-M06-2X/def2-SVP). The locally excited Ph_2 fragment is indicated by the red rectangle; b) NTO analysis of the excited state wavefunction showing dominant Ph_2* character (TD-M06-2X/def2-SVP).

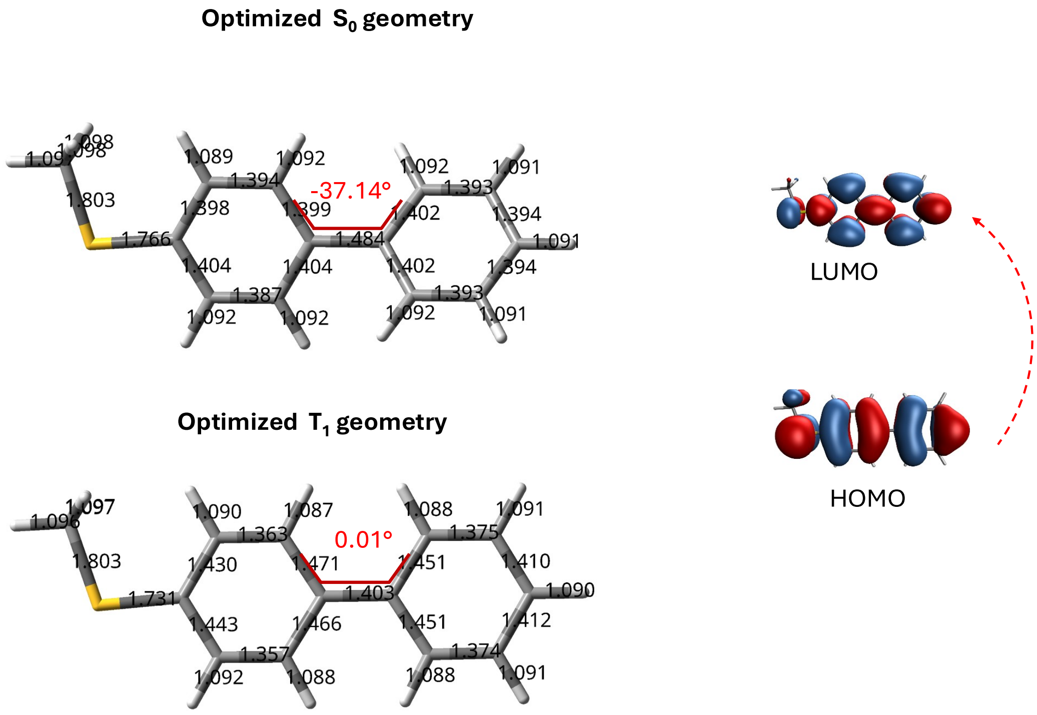

Figure S 24. Ph_2SMe: Optimized geometries of (top) the ground (M06-2X/def2-SVP), (bottom) the lowest triplet excited state (TD-M06-2X/def2-SVP); (right) the frontier molecular orbitals involved in the Ph_2* excited state.

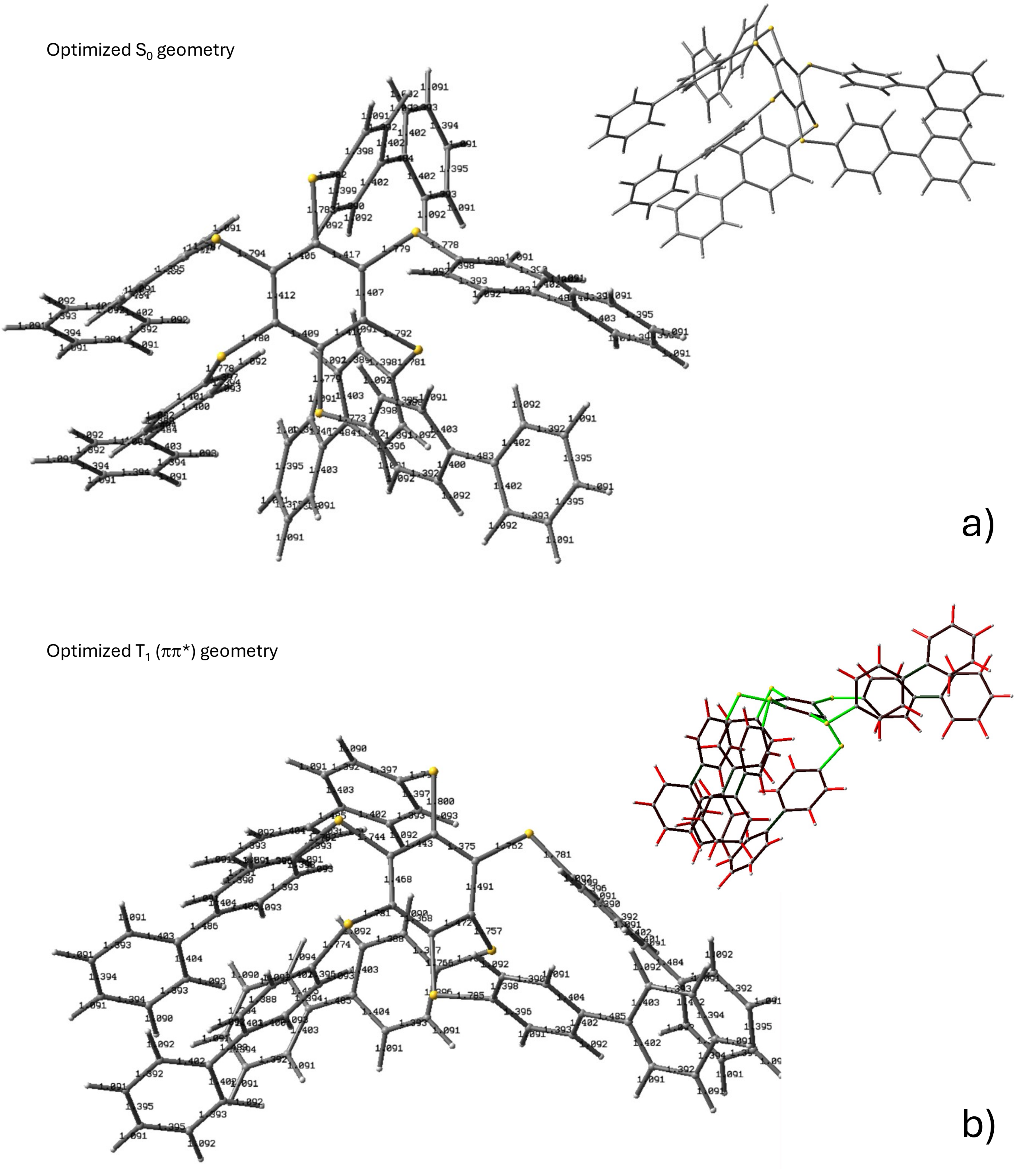

Figure S 25. A6-Ph₂, *ababbb* conformer: Optimized geometries of a) the ground (M06-2X/def2-SVP), b) the lowest triplet excited state with ππ* character (TD-M06-2X/def2-SVP).

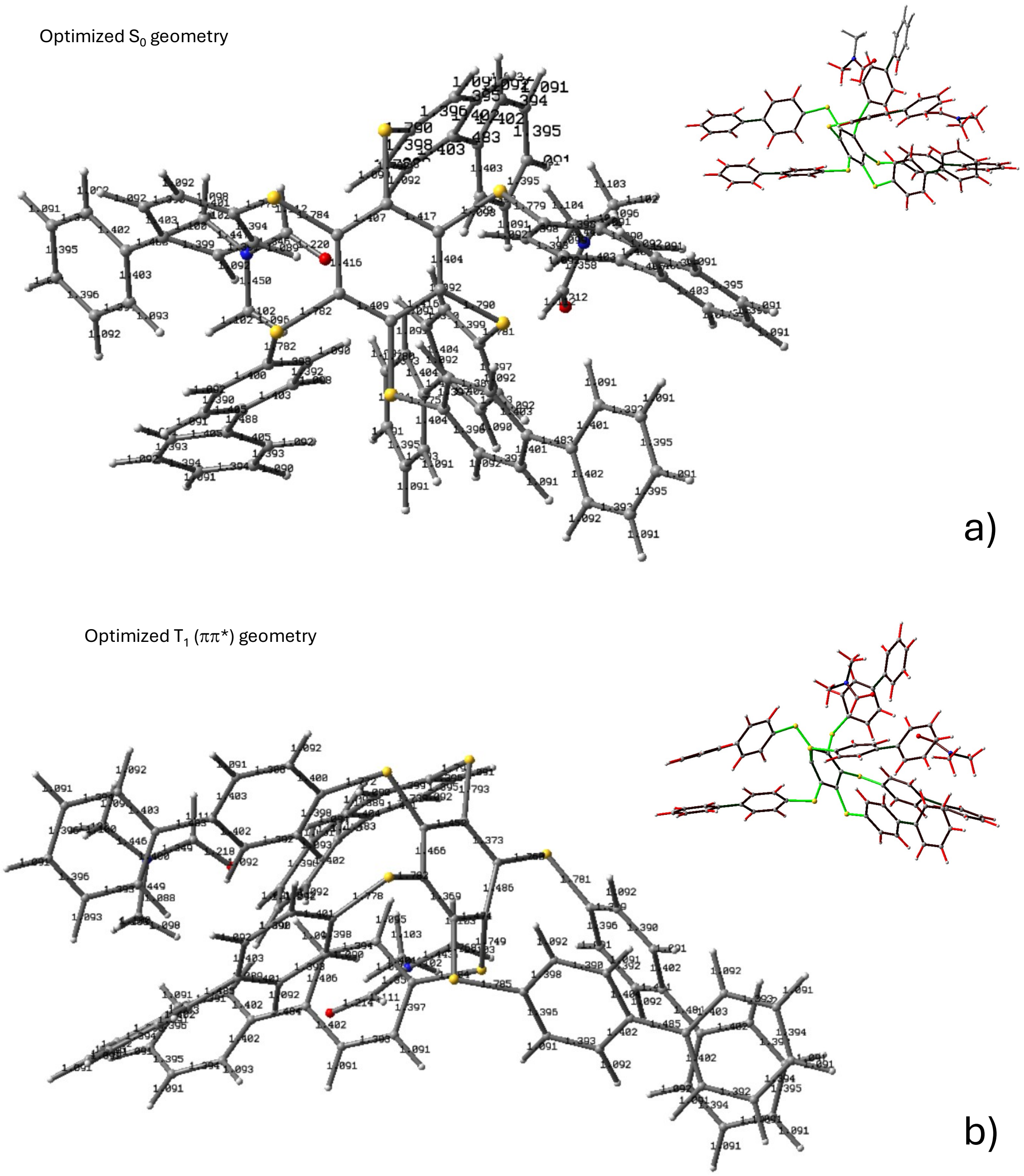

Figure S 26. A6-Ph₂, *ababbb* conformer: Optimized geometries, including 2 DMF solvent molecules, of a) the ground (M06-2X/def2-SVP), b) the lowest triplet excited state with $\pi\pi^*$ character (TD-M06-2X/def2-SVP).

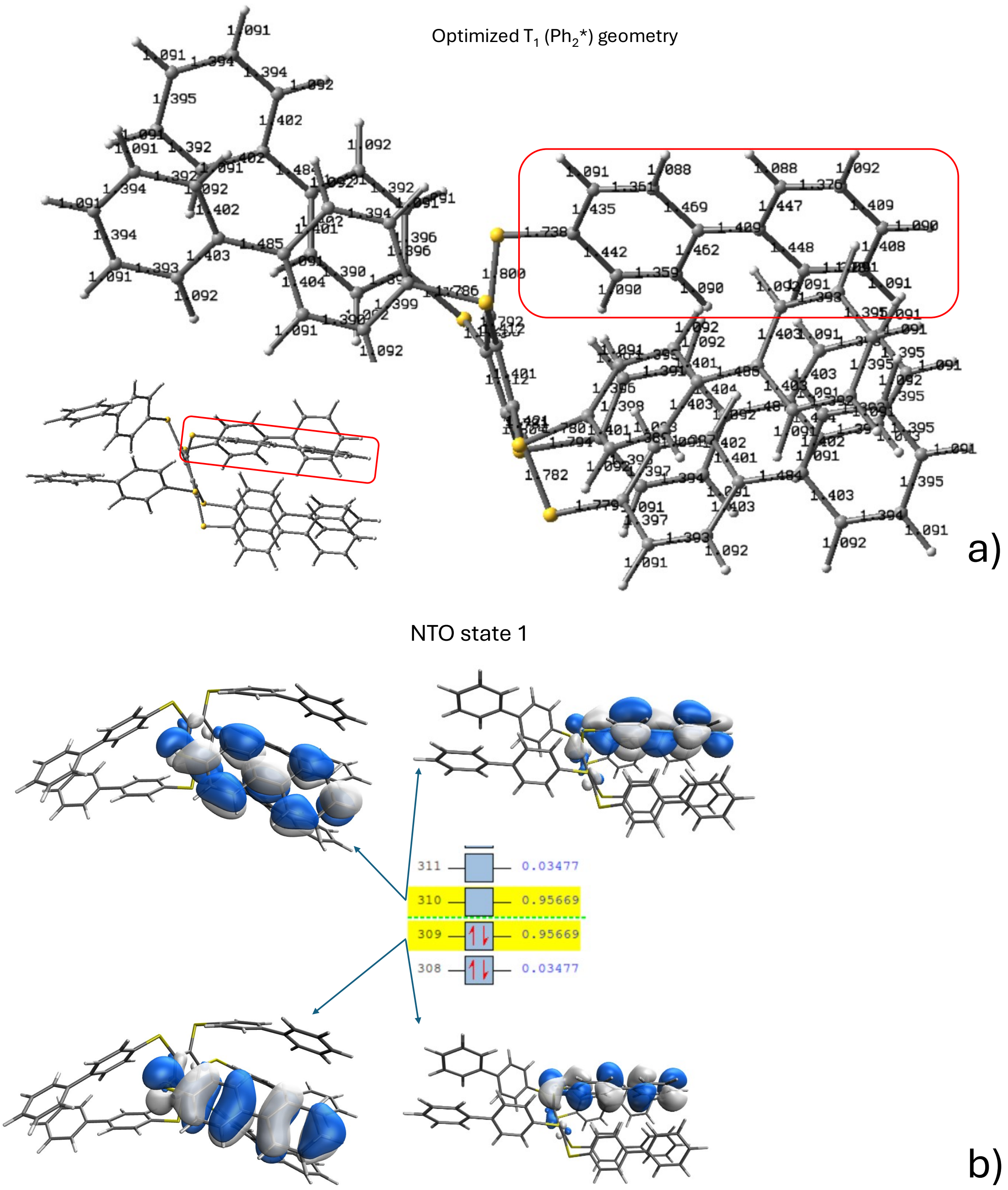

Figure S 27. A6-Ph$_2$, *ababbb* conformer: a) Optimized geometry of the lowest triplet state showing dominant Ph$_2$* character (TD-M06-2X/def2-SVP). The locally excited Ph$_2$ fragment is indicated by the red rectangle; b) NTO analysis of the excited state wavefunction showing dominant Ph$_2$* character (TD-M06-2X/def2-SVP).

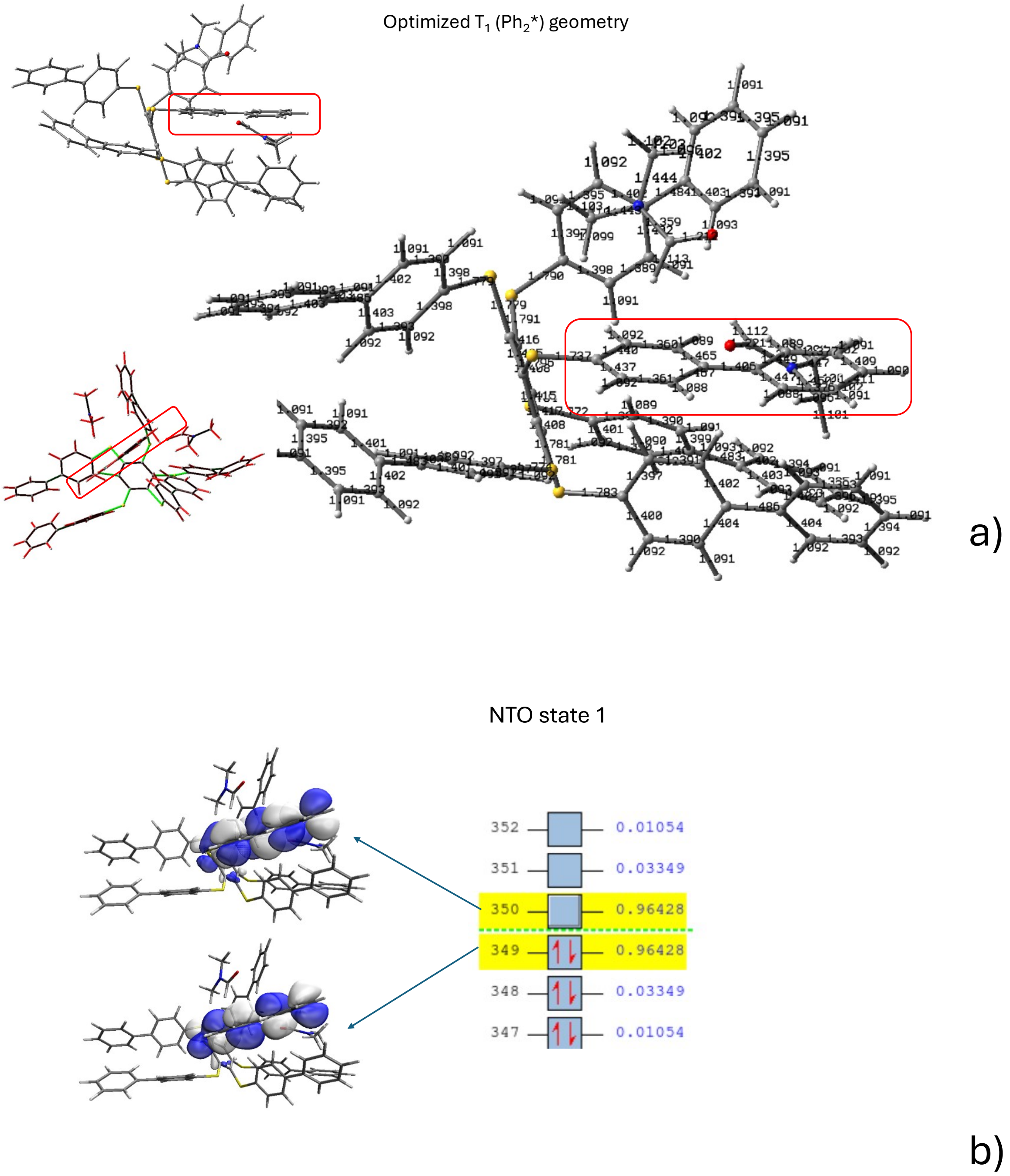

Figure S 28. A6-Ph₂, *ababbb* conformer including 2 DMF solvent molecules: a) Optimized geometry of the lowest triplet state showing dominant Ph_2* character (TD-M06-2X/def2-SVP). The locally excited Ph_2 fragment is indicated by the red rectangle; b) NTO analysis of the excited state wavefunction showing dominant Ph_2* character (TD-M06-2X/def2-SVP).

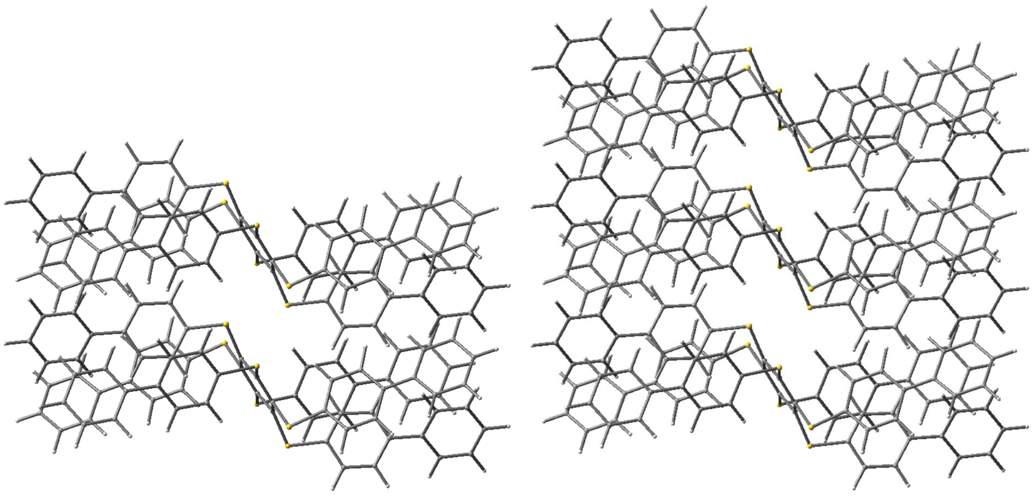

Figure S 29. A6-Ph$_2$, *aaabbb* conformer: dimer and trimer structures on which TD-M06-2X/def2-SVP calculations have been carried out. Results are collected in Table S6.

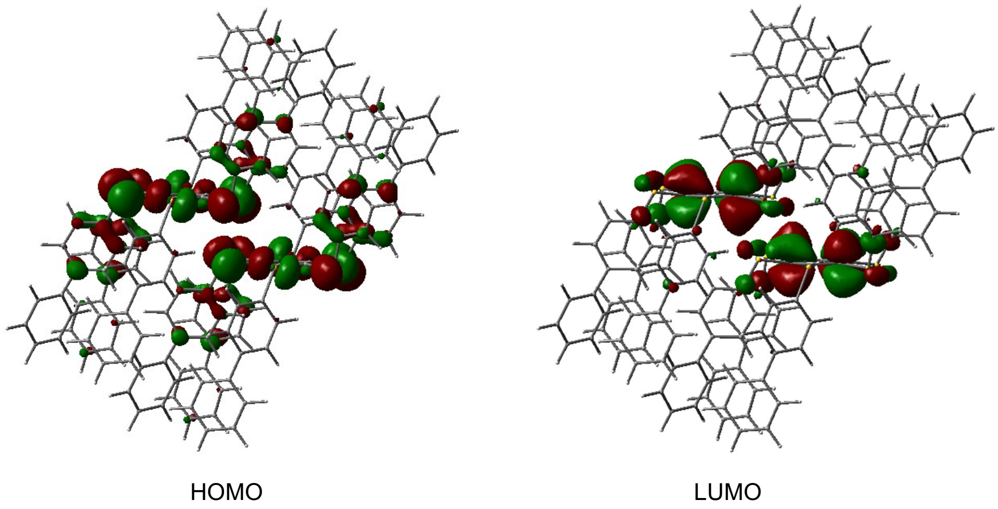

Figure S 30. A6-Ph$_2$, *aaabbb* conformer: dimer. Frontier molecular orbitals that dominate the $T_1(n\pi^*)$ state wavefunction. Exciton interactions are negligible due to modest orbital overlap.

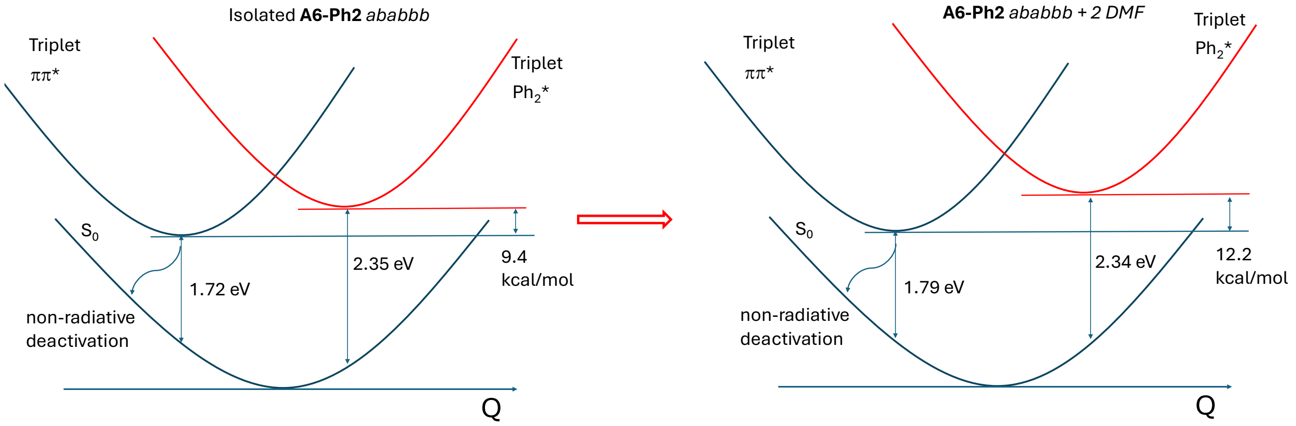

Figure S 31. A6-Ph2, *ababbb* conformer: schematic representation of the potential energy profiles of the two triplet excited states (ππ* and Ph2* character; TD-M06-2X/def2-SVP) and of the ground state (M06-2X/def2-SVP) for (left) the isolated molecule and (right) including two explicit DMF solvent molecules. Q is a displacement coordinate representing the multidimensional molecular distortion.

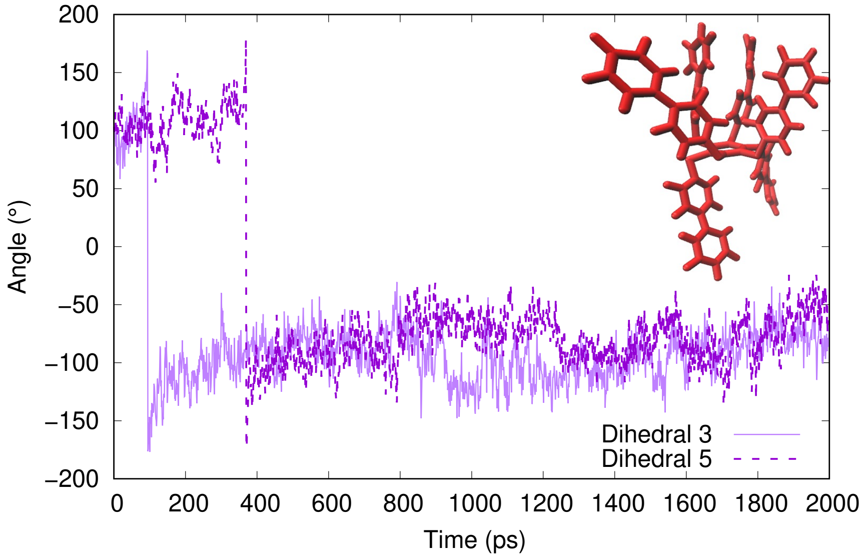

Figure S 32. The sharp dihedral angle change for two out of six dihedral angles during one of the MM3 MD simulations on a 5×5×5 supercell of **A6-Ph$_2$** molecules. Inset: the final conformer of the molecule reached after multiple angle switch.

SUPPORTING REFERENCES

(1) Tucker, J. H. R.; Gingras, M.; Brand, H.; Lehn, J.-M. Redox Properties of Polythiaarene Derivatives. A Novel Class of Electron Acceptors. *J. Chem. Soc. Perkin Trans. 2* **1997**, No. 7, 1303–1308. https://doi.org/10.1039/a608455i.

(2) Bergamini, G.; Fermi, A.; Botta, C.; Giovanella, U.; Di Motta, S.; Negri, F.; Peresutti, R.; Gingras, M.; Ceroni, P. A Persulfurated Benzene Molecule Exhibits Outstanding Phosphorescence in Rigid Environments: From Computational Study to Organic Nanocrystals and OLED Applications. *J. Mater. Chem. C* **2013**, *1* (15), 2717–2724. https://doi.org/10.1039/c3tc00878a.

(3) Fermi, A.; Bergamini, G.; Peresutti, R.; Marchi, E.; Roy, M.; Ceroni, P.; Gingras, M. Molecular Asterisks with a Persulfurated Benzene Core Are among the Strongest Organic Phosphorescent Emitters in the Solid State. *Dyes Pigm.* **2014**, *110*, 113–122. https://doi.org/10.1016/j.dyepig.2014.04.036.

(4) Li, X.; Wu, Q.; Bai, J.; Hou, S.; Jiang, W.; Tang, C.; Song, H.; Huang, X.; Zheng, J.; Yang, Y.; Liu, J.; Hu, Y.; Shi, J.; Liu, Z.; Lambert, C. J.; Zhang, D.; Hong, W. Structure-Independent Conductance of Thiophene-Based Single-Stacking Junctions. *Angew. Chem. Int. Ed.* **2020**, *59* (8), 3280–3286. https://doi.org/10.1002/anie.201913344.

(5) Koziakov, D.; Majek, M.; Jacobi von Wangelin, A. Metal-Free Radical Thiolations Mediated by Very Weak Bases. *Org. Biomol. Chem.* **2016**, *14* (48), 11347–11352. https://doi.org/10.1039/C6OB02276F.

(6) Sheldrick, G. M. SHELXT – Integrated Space-Group and Crystal-Structure Determination. *Acta Crystallogr. Sect. A Found. Adv.* **2015**, *71* (1), 3–8. https://doi.org/10.1107/S2053273314026370.

(7) Sheldrick, G. M. Crystal Structure Refinement with SHELXL. *Acta Crystallogr. Sect. C Struct. Chem.* **2015**, *71* (1), 3–8. https://doi.org/10.1107/S2053229614024218.

(8) Dolomanov, O. V.; Bourhis, L. J.; Gildea, R. J.; Howard, J. A. K.; Puschmann, H. OLEX2 : A Complete Structure Solution, Refinement and Analysis Program. *J. Appl. Crystallogr.* **2009**, *42* (2), 339–341. https://doi.org/10.1107/S0021889808042726.

(9) Altomare, A.; Cuocci, C.; Giacovazzo, C.; Moliterni, A.; Rizzi, R.; Corriero, N.; Falcicchio, A. EXPO2013 : A Kit of Tools for Phasing Crystal Structures from Powder Data. *J. Appl. Crystallogr.* **2013**, *46* (4), 1231–1235. https://doi.org/10.1107/S0021889813013113.

(10) Altomare, A.; Giacovazzo, C.; Guagliardi, A.; Moliterni, A. G. G.; Rizzi, R.; Werner, P.-E. New Techniques for Indexing: N-TREOR in EXPO. *J. Appl. Crystallogr.* **2000**, *33* (4), 1180–1186. https://doi.org/10.1107/S0021889800006427.

(11) Rappe, A. K.; Casewit, C. J.; Colwell, K. S.; Goddard, W. A.; Skiff, W. M. UFF, a Full Periodic Table Force Field for Molecular Mechanics and Molecular Dynamics Simulations. *J. Am. Chem. Soc.* **1992**, *114* (25), 10024–10035. https://doi.org/10.1021/ja00051a040.

(12) MOPAC2016, Http://OpenMOPAC.Net/, Version Nn.Nnn, J.J.P. Stewart, Stewart Computational Chemistry, Colorado Springs, CO, USA.

(13) Macrae, C. F.; Sovago, I.; Cottrell, S. J.; Galek, P. T. A.; McCabe, P.; Pidcock, E.; Platings, M.; Shields, G. P.; Stevens, J. S.; Towler, M.; Wood, P. A. Mercury 4.0 : From Visualization to Analysis, Design and Prediction. *J. Appl. Crystallogr.* **2020**, *53* (1), 226–235. https://doi.org/10.1107/S1600576719014092.

(14) Crosby, G. A.; Demas, J. N. Measurement of Photoluminescence Quantum Yields. Review. *J. Phys. Chem.* **1971**, *75* (8), 991–1024. https://doi.org/10.1021/j100678a001.

(15) De Mello, J. C.; Wittmann, H. F.; Friend, R. H. An Improved Experimental Determination of External Photoluminescence Quantum Efficiency. *Adv. Mater.* **1997**, *9* (3), 230–232. https://doi.org/10.1002/adma.19970090308.

(16) Fermi, A.; d'Agostino, S.; Dai, Y.; Brunetti, F.; Negri, F.; Gingras, M.; Ceroni, P. Unravelling the Role of Structural Factors in the Luminescence Properties of Persulfurated Benzenes. *Chem. Eur. J.* **2024**, *30* (43), e202401768. https://doi.org/10.1002/chem.202401768.

(17) Grimme, S.; Antony, J.; Ehrlich, S.; Krieg, H. A Consistent and Accurate Ab Initio Parametrization of Density Functional Dispersion Correction (DFT-D) for the 94 Elements H-Pu. *J. Chem. Phys.* **2010**, *132* (15), 154104. https://doi.org/10.1063/1.3382344.

(18) Frisch, M. J.; Trucks, G. W.; Schlegel, H. B.; Scuseria, G. E.; Robb, M. A.; Cheeseman, J. R.; Scalmani, G.; Barone, V.; Petersson, G. A.; Nakatsuji, H.; Li, X.; Caricato, M.; Marenich, A. V.; Bloino, J.; Janesko, B. G.; Gomperts, R.; Mennucci, B.; Hratchian, H. P.; Ortiz, J. V.; Izmaylov, A. F.; Sonnenberg, J. L.; Williams-Young, D.; Ding, F.; Lipparini, F.; Egidi, F.; Goings, J.; Peng, B.; Petrone, A.; Henderson, T.; Ranasinghe, D.; Zakrzewski, V. G.; Gao, J.; Rega, N.; Zheng, G.; Liang, W.; Hada, M.; Ehara, M.; Toyota, K.; Fukuda, R.; Hasegawa, J.; Ishida, M.; Nakajima, T.; Honda, Y.; Kitao, O.; Nakai, H.; Vreven, T.; Throssell, K.; Montgomery, J. A., J.; Peralta, J. E.; Ogliaro, F.; Bearpark, M. J.; Heyd, J. J.; Brothers, E. N.; Kudin, K. N.; Staroverov, V. N.; Keith, T. A.; Kobayashi, R.; Normand, J.; Raghavachari, K.; Rendell, A. P.; Burant, J. C.; Iyengar, S. S.; Tomasi, J.; Cossi, M.; Millam, J. M.; Klene, M.; Adamo, C.; Cammi, R.; Ochterski, J. W.; Martin, R. L.; Morokuma, K.; Farkas, O.; Foresman, J. B.; Fox, D. J. Gaussian 16 Revision A.03. *Revision A.03, Gaussian, Inc., Wallingford CT,*. 2016.

(19) Dapprich, S.; Komáromi, I.; Byun, K. S.; Morokuma, K.; Frisch, M. J. A New ONIOM Implementation in Gaussian98. Part I. The Calculation of Energies, Gradients, Vibrational Frequencies and Electric Field Derivatives. *J. Mol. Struct. THEOCHEM* **1999**, *461–462*, 1–21. https://doi.org/10.1016/S0166-1280(98)00475-8.

(20) Rappe, A. K.; Goddard, W. A. Charge Equilibration for Molecular Dynamics Simulations. *J. Phys. Chem.* **1991**, *95* (8), 3358–3363. https://doi.org/10.1021/j100161a070.

(21) Mayo, S. L.; Olafson, B. D.; Goddard, W. A. DREIDING: A Generic Force Field for Molecular Simulations. *J. Phys. Chem.* **1990**, *94* (26), 8897–8909. https://doi.org/10.1021/j100389a010.

(22) Lii, J. H.; Allinger, N. L. Molecular Mechanics. The MM3 Force Field for Hydrocarbons. 2. Vibrational Frequencies and Thermodynamics. *J. Am. Chem. Soc.* **1989**, *111* (23), 8566–8575. https://doi.org/10.1021/ja00205a002.

(23) Jorgensen, W. L.; Ghahremanpour, M. M.; Saar, A.; Tirado-Rives, J. OPLS/2020 Force Field for Unsaturated Hydrocarbons, Alcohols, and Ethers. *J. Phys. Chem. B* **2024**, *128* (1), 250–262. https://doi.org/10.1021/acs.jpcb.3c06602.

(24) Rackers, J. A.; Wang, Z.; Lu, C.; Laury, M. L.; Lagardère, L.; Schnieders, M. J.; Piquemal, J.-P.; Ren, P.; Ponder, J. W. Tinker 8: Software Tools for Molecular Design. *J. Chem. Theory Comput.* **2018**, *14* (10), 5273–5289. https://doi.org/10.1021/acs.jctc.8b00529.

(25) Thompson, A. P.; Aktulga, H. M.; Berger, R.; Bolintineanu, D. S.; Brown, W. M.; Crozier, P. S.; in 't Veld, P. J.; Kohlmeyer, A.; Moore, S. G.; Nguyen, T. D.; Shan, R.; Stevens, M. J.; Tranchida, J.; Trott, C.; Plimpton, S. J. LAMMPS - a Flexible Simulation Tool for Particle-Based Materials Modeling at the Atomic, Meso, and Continuum Scales. *Comput. Phys. Commun.* **2022**, *271*, 108171. https://doi.org/10.1016/j.cpc.2021.108171.

(26) Manz, T. A.; Limas, N. G. Introducing DDEC6 Atomic Population Analysis: Part 1. Charge Partitioning Theory and Methodology. *RSC Adv.* **2016**, *6* (53), 47771–47801. https://doi.org/10.1039/C6RA04656H.

(27) Tkatchenko, A.; Scheffler, M. Accurate Molecular Van Der Waals Interactions from Ground-State Electron Density and Free-Atom Reference Data. *Phys. Rev. Lett.* **2009**, *102* (7), 073005. https://doi.org/10.1103/PhysRevLett.102.073005.

(28) Lin, K.-H.; Paterson, L.; May, F.; Andrienko, D. Glass Transition Temperature Prediction of Disordered Molecular Solids. *npj Comput. Mater.* **2021**, *7* (1), 179. https://doi.org/10.1038/s41524-021-00647-w.

(29) Watkins, E. K.; Jorgensen, W. L. Perfluoroalkanes: Conformational Analysis and Liquid-State Properties from Ab Initio and Monte Carlo Calculations. *J. Phys. Chem. A* **2001**, *105* (16), 4118–4125. https://doi.org/10.1021/jp004071w.
